# Targeting conserved domains of hypoxia-inducible factors for cancer therapy

**DOI:** 10.1084/jem.20251009

**Published:** 2026-04-02

**Authors:** Shaima Salman, Tina Y. Huang, Yousang Hwang, Anmol Kumar, Emmanuel Datan, Elizabeth E. Wicks, Ellen Cho, Sophia N. Lee, Chelsey Chen, Ying-Jie Peng, Dominic Dordai, Oscar Li, Daiana Drehmer, Yajing Lyu, Yongkang Yang, Walter Jackson, Nanduri R. Prabhakar, Alexander D. MacKerell, Gregg L. Semenza

**Affiliations:** 1 Department of Genetic Medicine, Institute for Cell Engineering, and Armstrong Oxygen Biology Research Center, Johns Hopkins University School of Medicine, Baltimore, MD, USA; 2 Department of Pharmaceutical Sciences, University of Maryland Computer-Aided Drug Design Center, University of Maryland School of Pharmacy, Baltimore, MD, USA; 3 Institute for Integrative Physiology, University of Chicago, Chicago, IL, USA; 4 Johns Hopkins University, Baltimore, MD, USA; 5 Johns Hopkins Kimmel Cancer Center, Baltimore, MD, USA

## Abstract

Hypoxia-inducible factors 1 and 2 (HIF-1/2) function as master regulators of cancer progression by regulating angiogenesis, cancer stem cell specification, epithelial–mesenchymal transition, immune evasion, tissue invasion, and metastasis. We utilized computer-aided drug discovery and cell-based reporter assays to identify dual HIF-1/2 inhibitors, which bind directly to highly conserved domains of HIF-1α and HIF-2α, disrupt dimerization with HIF-1β, and trigger proteasomal degradation, thereby inhibiting HIF-1/2 transcriptional activity. These inhibitors and derivative compounds block growth and vascularization of breast, colorectal, head/neck, melanoma, and prostate tumors as monotherapy, and overcome resistance to anti-CTLA-4 or anti-PD-1 immunotherapy, with an aggregate complete response rate of over 50%, through reprogramming of the tumor immune cell microenvironment. Compared with the HIF-2–selective inhibitors belzutifan and PT2385, dual HIF-1/2 inhibitor 1.21S9N showed superior activity against breast and colorectal cancer models, respectively. PT2385 caused breathing abnormalities, whereas 1.21S9N did not. The drugs are orally bioavailable, and no safety concerns were identified even after extended or supratherapeutic dosing.

## Teaser

Hypoxia-inducible factors 1 and 2 (HIF-1/2) play critical roles in cancer progression. We have developed the first dual HIF-1/2 inhibitors that bind to the most highly conserved domains of both proteins, show activity against a wide range of cancers, are orally bioavailable, and markedly improve responses to immune checkpoint blockade in mouse tumor models.

## Graphical Abstract

**Figure:**
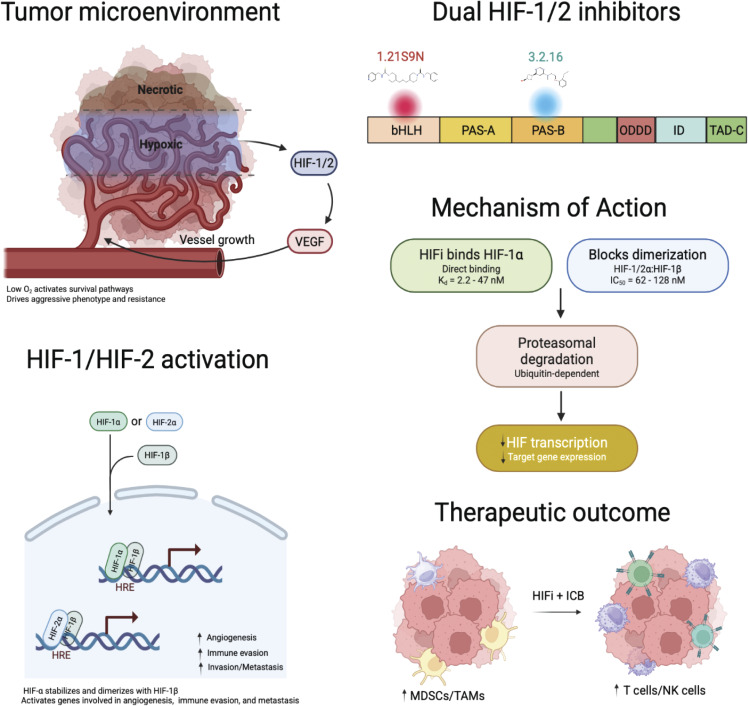

## Introduction

Hypoxia, defined as decreased O_2_ availability, is a common feature of many pathological conditions, including cancer, ischemic cardiovascular disease, and inflammatory disorders (Eltzschig and Carmeliet, 2011; Giaccia et al., 2003; Semenza, 2012a; Yuan et al., 2024). In solid tumors, dysregulated cancer cell proliferation and recruitment of immune cells, coupled with the generation of blood vessels that are structurally and functionally abnormal, leads to a mismatch of O_2_ supply and demand, including regions with severe intratumoral hypoxia (Bertout et al., 2008; Vaupel et al., 2007; Wicks and Semenza, 2022). Direct measurement by Eppendorf microelectrode revealed that in normal breast tissue, the median pO_2_ was 65 mmHg and no value <12.5 mmHg was recorded, whereas in advanced breast cancers (BrCa), the median pO_2_ was 10 mmHg (Vaupel et al., 2007). In prostate cancer, a median pO_2_ value of 2.4 mmHg was reported (Movsas et al., 2002). The presence of intratumoral hypoxia is independent of tumor grade, stage, or size and is a predictor of metastasis, treatment failure, and patient mortality (Vaupel et al., 2007).

In response to hypoxia, cells in most metazoans activate a transcriptional pathway mediated by hypoxia-inducible factors (HIFs), which play a crucial role in adaptation to low O_2_ levels (Semenza, 2012a; Yuan et al., 2024). HIFs are heterodimeric transcription factors composed of an O_2_-sensitive HIF-α subunit (HIF-1α, HIF-2α, or HIF-3α) and a constitutively expressed HIF-1β subunit (Wang et al., 1995; Tian et al., 1997; Yuan et al., 2024). The amino-terminal half of both subunits consists of highly conserved basic–helix–loop–helix (bHLH) and Per-Arnt-Sim (PAS) domains that are required for dimerization and DNA binding (Jiang et al., 1996; Wang et al., 1995). The carboxy-terminal half of the HIF-α subunits consists of O_2_-dependent degradation and transactivation domains (Jiang et al., 1997; Pugh et al., 1997). Under normoxic conditions, HIF-α subunits are subjected to O_2_-dependent prolyl hydroxylation and binding of the VHL protein, which targets HIF-α subunits for ubiquitination and proteasomal degradation (Epstein et al., 2001; Maxwell et al., 1999). When O_2_ levels decrease, hydroxylation is inhibited, and HIF-α subunits dimerize with HIF-1β, bind to hypoxia response elements (HREs), and activate transcription of target genes (Yuan et al., 2024). Many oncogene gain-of-function and tumor suppressor loss-of-function mutations increase HIF activity in an O_2_-independent manner (Semenza, 2010).

The expression of HIF target genes (Winter et al., 2007) and the expression of HIF-1α or HIF-2α protein (Wicks and Semenza, 2022) in tumor biopsies are associated with patient mortality in many cancers, reflecting the role of HIFs in directing tumor vascularization, metabolic reprogramming, epithelial–mesenchymal transition, cell motility, extracellular matrix remodeling, cancer stem cell specification, immune evasion, invasion, metastasis, and treatment failure (Noman et al., 2019; Rankin et al., 2016; Sitkovsky et al., 2014; Wicks and Semenza, 2022). The role of HIF-3α in cancer biology has not been well established, and in many cancer cell lines, it is not expressed at all. Although HIF-1α and HIF-2α are both O_2_-regulated and show high sequence similarity in the bHLH and PAS domains, they are often expressed in different tumor niches and have nonoverlapping roles in various human cancer and associated stromal cells, with HIF-1α mediating acute hypoxic responses and glycolysis, and HIF-2α driving chronic hypoxic responses and MYC-mediated proliferation (Cowman and Koh, 2022; Keith et al., 2011). Thus, dual HIF-1/2 inhibition strategies are needed to achieve effective and sustained antitumor responses.

The interaction of immune checkpoint receptors CTLA-4 and PD-1, which are expressed on T cells and natural killer (NK) cells, with their cognate ligands CD80/86 and PD-L1, respectively, which are expressed on antigen-presenting cells and cancer cells, induces T and NK cell exhaustion or apoptosis, and immune checkpoint blockade (ICB) by antibodies against CTLA-4 (α-CTLA-4), PD-1 (α-PD-1), or PD-L1 has been approved by the US Food and Drug Administration (FDA) for the treatment of many cancers (Topalian et al., 2015; Topalian et al., 2020; Sharma et al., 2021). Although ICB can lead to tumor eradication, it is ineffective in the majority of patients, because T and NK cells have been excluded from the tumor or are present but do not respond to ICB because the cancer has employed other mechanisms of immune evasion (Crespo et al., 2013; Zou, 2005). Hypoxia alters the tumor immune cell microenvironment (TIME) to favor immunosuppression over antitumor immunity (Chouaib et al., 2012; Noman et al., 2019; Semenza, 2021; Sitkovsky et al., 2014). HIF-1 mediates increased expression of PD-L1, which binds to PD-1 on T and NK cells to induce exhaustion or apoptosis (Barsoum et al., 2014; Noman et al., 2014), and CD73, an extracellular enzyme that generates adenosine, which also binds to cognate receptors on T and NK cells to induce exhaustion or apoptosis (Sitkovsky et al., 2014; Synnestvedt et al., 2002). Cancers expressing both CD73 and PD-L1 are resistant to therapies targeting either one.

Belzutifan (PT2977) binds selectively to the PAS-B subdomain of HIF-2α to block dimerization with HIF-1β. It received initial approval by the FDA for VHL disease-associated renal cell carcinoma (RCC), central nervous system hemangioblastomas, and pancreatic neuroendocrine tumors, and subsequent approvals have included patients with advanced or metastatic RCC (Fallah et al., 2022, 2024; Jonasch et al., 2021). However, it is unknown whether selective inhibition of HIF-2 will benefit patients with other more common types of cancer. Given the distinct roles of HIF-1 and HIF-2 in cancer progression, dual HIF-1/2 inhibition presents a promising therapeutic strategy, particularly for cancer types with a known propensity for intratumoral hypoxia and/or resistance to conventional therapy (Semenza, 2012b; Wicks and Semenza, 2022; Wilson and Hay, 2011; Yuan et al., 2024). Several HIF inhibitors, including YC-1 (Yeo et al., 2003), echinomycin (Kong et al., 2005), PX-478 (Koh et al., 2008), IDF-1174 (Shen et al., 2022), and 32-134D (Salman et al., 2022), have antitumor activity as monotherapy and potentiate the effect of ICB in mouse models (Bailey et al., 2022; Luo et al., 2022; Ma et al., 2022; Salman et al., 2022; Shen et al., 2022). However, unlike belzutifan and the related compound PT2385 (Wallace et al., 2016), none of these other inhibitors bind directly to HIFs or are in clinical use. Recent structural studies of HIFs and related bHLH-PAS transcription factors have detailed ligand-accessible PAS-B cavities, quaternary architectures of HIF heterodimers, and allosteric modulation of dimer stability and DNA binding by small molecules that suggest the feasibility of targeting HIF-1/2 bHLH/PAS interfaces more broadly (Wu et al., 2019; Zhuang et al., 2024). Here, we describe small-molecule dual HIF-1/2 inhibitors (HIFi) that bind directly to the most highly conserved domains of HIF-1/2α, block dimerization with HIF-1β, and cause HIF-1/2α degradation.

## Results

### Identification of candidate ligand binding sites in HIF-2α

**Figure 1.**
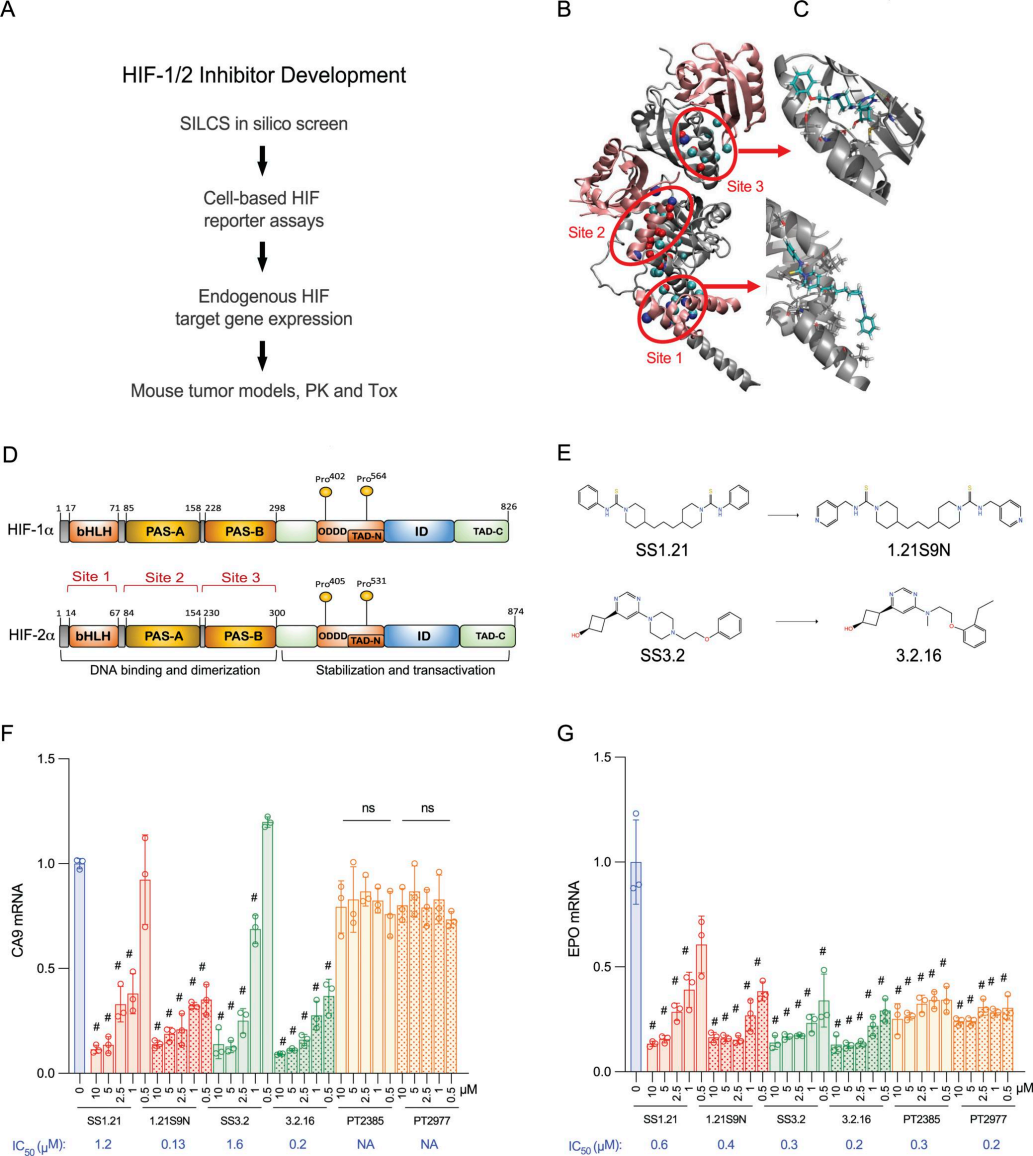
**Discovery and optimization of dual** **HIFi** **. (A)** Overview of the workflow is presented. **(B)** Crystal structure of HIF-2α–HIF-1β was analyzed using SILCS, which identified three potential ligand binding sites. **(C)** Models of site 1 and site 3 occupancy by SS1.21 and SS3.2, respectively, are shown. **(D)** Highly conserved domain organization of HIF-1α and HIF-2α is shown with location of candidate ligand binding sites indicated by red brackets. **(E)** Chemical structures of dual HIFi are shown. SS1.21 and derivative compound 1.21S9N bind to site 1, whereas SS3.2 and derivative compound 3.2.16 bind to site 3. **(F and G)** Expression of CA9 (F) and EPO (G) mRNA was analyzed in Hep3B cells incubated for 24 h at 20% or 1% O_2_ with vehicle, or at 1% O_2_ with the indicated HIF inhibitor at the indicated concentration (μM), and derived IC_50_ values are shown. Results are presented as the mean ± SD (*n* = 3). ^#^P < 0.05 versus 1% O_2_ (vehicle); ns, no significant difference versus 1% O_2_ (vehicle); two-way ANOVA with Dunnett’s multiple comparisons post-test.

Computer-aided drug discovery using the crystal structure of the HIF-2α:HIF-1β complex (PDB: 4ZP4; Wu et al., 2015) and the site identification by ligand competitive saturation (SILCS) approach (Guvench and MacKerell, 2009) was performed. SILCS simulations produce three-dimensional distributions of grid-free energies (GFEs) of functional groups throughout the protein termed FragMaps (MacKerell et al., 2020), which were used to identify three potential ligand binding sites in the bHLH domain (site 1), PAS-A subdomain (site 2), and PAS-B subdomain (site 3) of HIF-2α (Fig. 1, A–D). FragMaps were utilized to generate pharmacophore features, which identify the types of functional groups and spatial relationships between those features that drug-like molecules should possess in order to bind at each site (Fig. 1 B). Virtual database screening was performed by selecting pharmacophore hypotheses that each contained a subset of three or four out of the total number of features at each site in various spatial relationships (see Materials and methods). These pharmacophore hypotheses were individually screened against an in-house database containing 768,466 compounds, with ranking performed using the SILCS Monte Carlo approach. The top 100 candidate ligands at each site with the lowest ligand GFE (LGFE) values were chosen for further evaluation.

### Identification of compounds that inhibit HIF-1/2 transcriptional activity

Of the 300 candidate compounds, 293 were commercially available and screened for inhibition of HIF-1 transcriptional activity in Hep3B-c1 cells, which are stably transfected with: HIF-1–dependent reporter plasmid p2.1, in which firefly luciferase (FLuc) coding sequences are downstream of the *ENO1* HRE and a basal SV40 promoter; and control reporter plasmid pSV-RL, in which *Renilla* luciferase (RLuc) coding sequences are downstream of the SV40 promoter only, such that the FLuc/RLuc ratio served as a measure of HIF-1 transcriptional activity (Salman et al., 2022). Alternatively, we transfected Hep3B cells with: a HIF-2α expression vector; control reporter pSV-RL; and pEPO-ProEn-FL, a reporter containing FLuc coding sequences under the control of *EPO* promoter and enhancer sequences, such that the FLuc/RLuc ratio served as a measure of HIF-2 activity (Dioum et al., 2009). Cells were exposed to compounds and incubated at 20% or 1% O_2_ for 24 h. Compounds that decreased the FLuc/RLuc ratio by >50% at low micromolar concentrations were tested for their effect on the hypoxia-induced expression of endogenous CA9 and EPO mRNA, which is mediated by HIF-1 and HIF-2, respectively.

**Figure S1.**
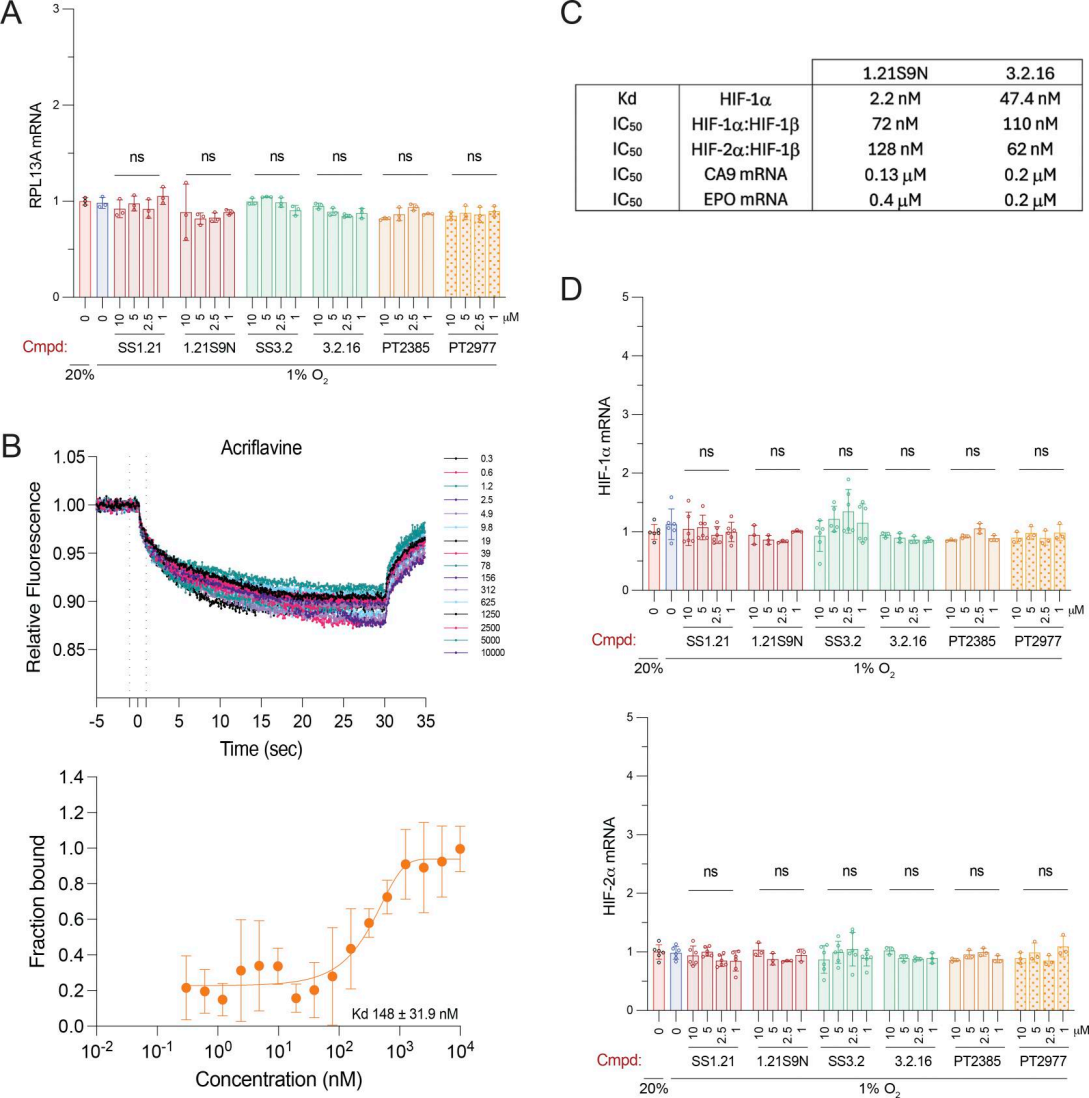
**Molecular analyses** **.** Related to Figs. 1, 2, and 3. **(A)** Hep3B cells were treated with 0–10 μM of the indicated dual HIF inhibitor (SS1.21, 1.21S9N, SS3.2, or 3.2.16) or HIF-2–selective inhibitor (PT2385 or PT2977) at 20% or 1% O_2_ for 24 h, and RPL13A mRNA expression was determined by RT-qPCR assays. Data are presented as the mean + SD (*n* = 3). ns, no significant difference versus vehicle-treated cells; two-way ANOVA with Dunnett’s multiple comparisons post-test. **(B)** Binding of acriflavine to fluorophore-tagged recombinant human HIF-1α was analyzed by MST. Data are presented as relative fluorescence at each concentration of added compound (nM; upper panel) and fraction bound with derived K_d_ estimate (mean ± SD, *n* = 4; lower panel). **(C)** Dual HIFi were characterized by: direct binding to purified HIF-1α using MST; inhibition of HIF-1α or HIF-2α dimerization with HIF-1β in cell lysates; and inhibition of HIF-1 target gene *CA9* and HIF-2 target gene *EPO* expression in hypoxic Hep3B cells. **(D)** HIF-α mRNA expression was determined by RT-qPCR assays. Data are presented as the mean +/− SD (*n* = 3–6). ns, no significant difference versus vehicle-treated cells; two-way ANOVA with Dunnett’s multiple comparisons post-test.

The candidate site 1 ligand SS1.21 (4,4′-(propane-1,3-diyl)bis(*N*-phenylpiperidine-1-carbothioamide)), which was identified in both the HIF-1 and HIF-2 reporter assays, and site 3 ligand SS3.2 (cis-3-(6-(4-(2-phenoxyethyl)piperazin-1-yl)pyrimidin-4-yl)cyclobutan-1-ol), which was identified in the HIF-2 reporter assay, were found to inhibit endogenous HIF-1/2 target gene expression in Hep3B cells (Fig. 1, A–G). In agreement with the reporter assays, the concentration for 50% inhibition (IC_50_) of the HIF-2 target gene *EPO* (0.3 μM) by SS3.2 was lower than for the HIF-1 target *CA9* (1.6 μM), whereas for SS1.21, the IC_50_ for *EPO* was 0.6 μM and for *CA9* was 1.2 μM (Fig. 1, F and G). With respect to *EPO* gene expression, the inhibitory activity of SS3.2 (0.3 μM) was comparable to that of the selective HIF-2 inhibitors PT2385 (0.3 μM) and PT2977 (0.2 μM), whereas the latter compounds did not inhibit *CA9* expression (Fig. 1, F and G). None of the compounds had any effect on RPL13A mRNA expression, which is neither hypoxia-induced nor HIF-regulated (Fig. S1 A).

SS1.21 has a high estimated log P = 6.1, indicative of hydrophobicity and poor aqueous solubility. We generated 83 derivative compounds with decreased log *P* and tested their HIF inhibitory activity, leading to identification of 1.21S9N (4,4′-(propane-1,3-diyl)bis(N-(pyridine-4-ylmethyl)piperidine-1-carbothioamide)) (Fig. 1, E–G). We also screened 346 compounds, which had structural similarity to SS1.21 or SS3.2 and were predicted to maintain interaction with the HIF-2α:HIF-1β complex, in the cell-based reporter assays, and identified 3.2.16 (cis-3-(6-((2-(2-ethylphenoxy)ethyl)(methyl)amino) pyrimidin-4-yl)cyclobutan-1-ol) (Fig. 1, E–G).

### Microscale thermophoresis (MST) reveals direct binding of 1.21S9N and 3.2.16 to HIF-1α

**Figure 2.**
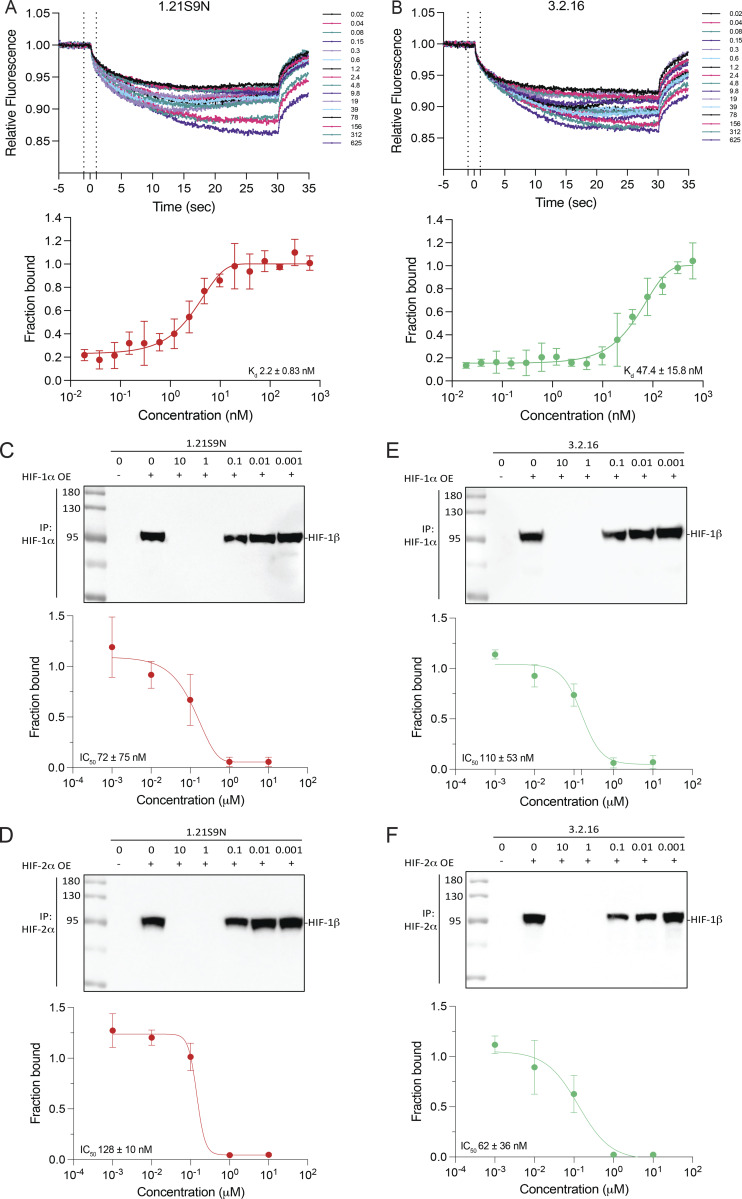
**Biophysical and molecular target engagement of dual** **HIFi** **. (A and B)** MST of fluorophore-tagged recombinant human HIF-1α following the addition of 1.21S9N (A) or 3.2.16 (B) was performed. Data are presented as relative fluorescence at each concentration of added compound (nM; upper panels) and fraction bound with derived K_d_ estimates (mean ± SD, *n* = 4; lower panels). **(C–F)** Lysates of Hep3B cells with OE of HIF-1α or HIF-2α were treated with HIFi and then incubated with lysate of cells with OE of HIF-1β. Concentration-dependent inhibition of HIF-1α–HIF-1β (C and E) or HIF-2α–HIF-1β (D and F) complex formation by 1.21S9N (C and D) or 3.2.16 (E and F) was analyzed by immunoprecipitation for HIF-α and immunoblot assay for HIF-1β. Representative Co-IP (upper panels) and fraction of HIF-1β bound (normalized to vehicle) with derived IC_50_ values (mean ± SD, *n* = 3; lower panels) are shown. OE, overexpression. Source data are available for this figure: SourceData F2.

We quantified dual HIFi binding to purified recombinant HIF-1α using MST. His_6_-tagged HIF-1α was complexed with a small-molecule N-nitriloacetic acid (NTA) fluorophore dye (Cy5-NT derivative) to enable label-free detection of the protein by His-Ni-NTA interaction as previously described (Woodfield et al., 2022). The HIF inhibitor acriflavine was found to bind with a K_d_ of 148 ± 31.9 nM (Fig. S1 B), which was comparable to previously published data using the same experimental design (Woodfield et al., 2022). Under the same conditions, MST traces and binding isotherms demonstrated that both 1.21S9N and 3.2.16 engage HIF-1α in a concentration-dependent manner. For 1.21S9N, the binding curve was well-fit by a single-site model, resulting in a K_d_ of 2.2 ± 0.83 nM (Fig. 2 A). 3.2.16 also bound HIF-1α with a single-site isotherm and a K_d_ of 47.4 ± 15.8 nM (Fig. 2 B). These results compare favorably with an MST study of PT2385 binding to HIF-2α in which the estimated K_d_ was 167 nM (Wu et al., 2019). Together, these data establish that 1.21S9N and 3.2.16 bind directly to purified HIF-1α with high affinity in vitro.

### Binding of HIFi to HIF-1α or HIF-2α blocks dimerization with HIF-1β

We next tested HIFi target engagement using a two-lysate co-immunoprecipitation (Co-IP) assay under nondenaturing conditions. HIF-1α, HIF-2α, and HIF-1β were overexpressed in separate cell populations and lysed independently. The HIF-1α or HIF-2α lysate was incubated with HIFi for 30 min at 4°C, HIF-1β lysate was added, HIF-1α or HIF-2α was immunoprecipitated, and HIF-1β Co-IP was quantified by the immunoblot assay. 1.21S9N inhibited the interaction of HIF-1α (Fig. 2 C) or HIF-2α (Fig. 2 D) with HIF-1β in a concentration-dependent manner, yielding a half-maximal effect (IC_50_) at 72 ± 75 nM and 128 ± 10 nM, respectively. 3.2.16 also decreased HIF-1α:HIF-1β (Fig. 2 E) and HIF-2α:HIF-1β (Fig. 2 F) complex recovery in a concentration-dependent manner, with an IC_50_ of 110 ± 53 nM and 62 ± 36 nM, respectively. These data are consistent with the MST data in demonstrating submicromolar target engagement for each HIFi (Fig. S1 C).

### Further delineation of mechanism of action and activity in many cancer cell types

**Figure 3.**
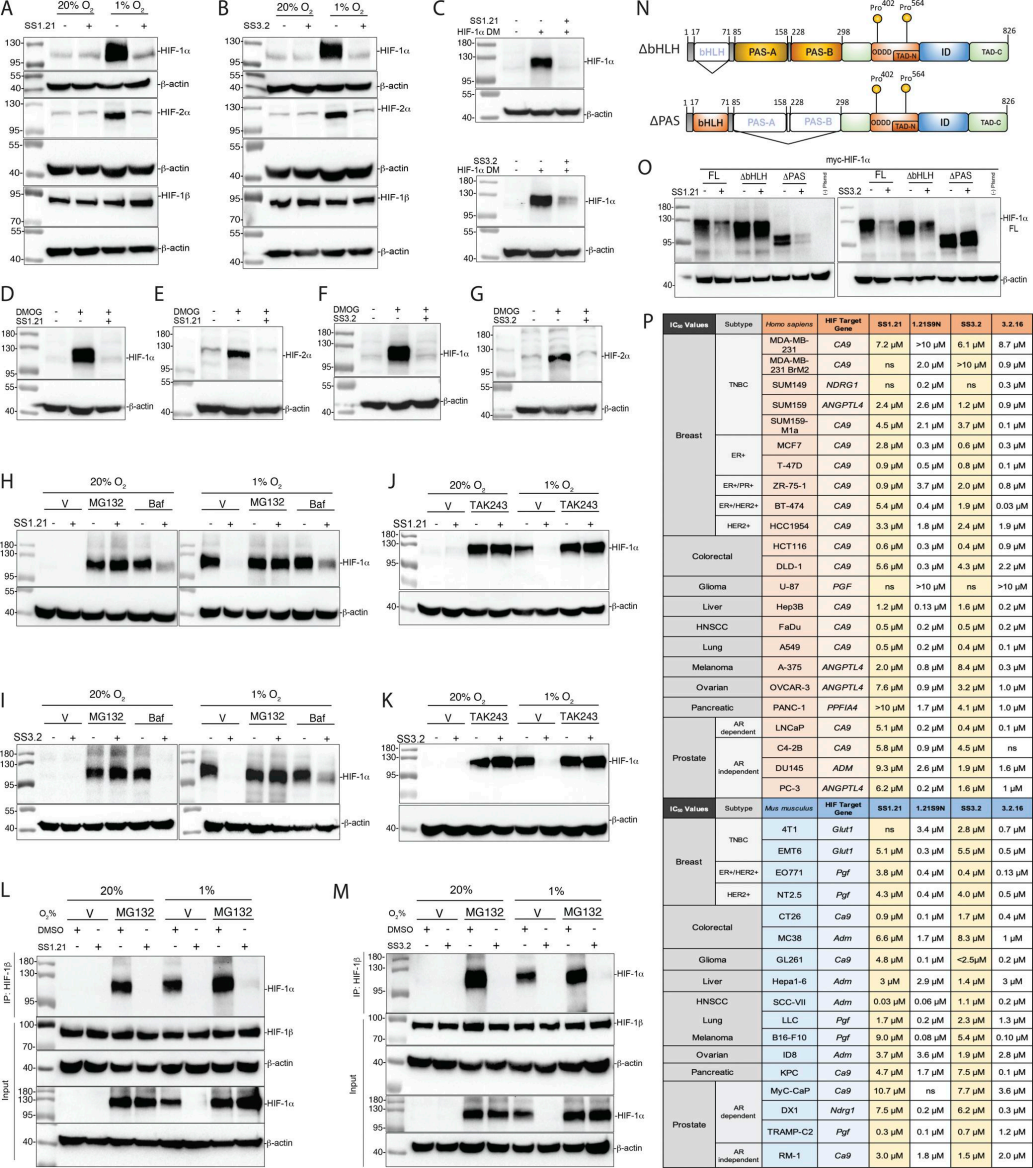
**SS1.21 and SS3.2 trigger degradation of HIF-1α and HIF-2α to inhibit HIF target gene expression. (A and B)** Hep3B cells were treated with 10-μM SS1.21 (A) or SS3.2 (B) at 20% or 1% O_2_ for 24 h, and immunoblot assays were performed. **(C)** Cells transfected with vector encoding HIF-1α-DM (P402A/P564A) were treated with vehicle, 10-μM SS1.21 (top), or SS3.2 (bottom) for 6 h. **(D–G)** Cells were treated with vehicle or 1 mM DMOG and vehicle or either 10-μM SS1.21 (D and E) or SS3.2 (F and G) for 6 h (D and F) or 24 h (E and G). **(H and I)** Cells were treated with 5-μM SS1.21 (H) or SS3.2 (I) in the presence of vehicle (V), 10-μM MG132, or 10-nM bafilomycin (Baf) at 20% or 1% O_2_ for 6 h. **(J and K)** Cells were treated with 5-μM SS1.21 (J) or SS3.2 (K) with V or 500-nM TAK243 for 6 h. **(L and M)** Cells were treated with 5-μM SS1.21 (L) or SS3.2 (M) with V or MG132, and immunoprecipitation was performed using an anti-HIF-1β antibody. Inputs and immunoprecipitates were subjected to immunoblot assays. **(N and O)** FL HIF-1α and deletion mutants (ΔbHLH and ΔPAS; N) were transiently expressed in Hep3B cells, which were treated with 5-μM SS1.21 (O, left) or SS3.2 (O, right). **(P)** IC_50_ values are shown for inhibition of target gene expression in cancer cells treated with indicated HIF inhibitor. All immunoblot assays were performed two to four times, and representative results are shown. See Fig. S2 A for data used to calculate IC_50_ values. Source data are available for this figure: SourceData F3.

Treatment of Hep3B cells with SS1.21 (Fig. 3 A) or SS3.2 (Fig. 3 B) blocked hypoxia-induced HIF-1α and HIF-2α protein accumulation. HIF-1β was constitutively expressed in all conditions (Fig. 3, A and B). Treatment with SS1.21 or SS3.2 had no significant effect on HIF-1α or HIF-2α mRNA levels (Fig. S1 D). SS1.21 or SS3.2 blocked accumulation of HIF-1α-DM, a hydroxylation-resistant form of HIF-1α in which Pro-402 and Pro-564 were doubly mutated to alanine (Fig. 3 C). SS1.21 (Fig. 3, D and E) or SS3.2 (Fig. 3, F and G) also inhibited HIF-1α and HIF-2α expression in cells treated with dimethyloxalylglycine, which blocks O_2_-dependent prolyl hydroxylation. MG132, which inhibits proteasome-dependent degradation, rescued HIF-1α expression in SS1.21 (Fig. 3 H)- or SS3.2 (Fig. 3 I)-treated cells, whereas bafilomycin, an inhibitor of lysosome-dependent degradation, did not (Fig. 3, H and I). Treatment with TAK243 to block protein ubiquitination also rescued HIF-1α expression in cells treated with SS1.21 (Fig. 3 J) or SS3.2 (Fig. 3 K).

Next, we analyzed whether treatment of intact cells with SS1.21 or SS3.2 would inhibit heterodimerization of HIF-1α and HIF-1β prior to protein degradation. To test this hypothesis, we performed Co-IP assays using lysates prepared from cells that were treated with SS1.21 or SS3.2 in the presence of MG132. HIF-1α was detected in lysates of MG132-treated cells exposed to 20% or 1% O_2_, either in the presence or in the absence of SS1.21 (Fig. 3 L) or SS3.2 (Fig. 3 M); however, HIF-1α co-immunoprecipitated with HIF-1β only in the absence of SS1.21 or SS3.2.

We hypothesized that if binding of SS1.21 or SS3.2 to site 1 or site 3, respectively, triggers HIF-1α degradation, then deletion of the bHLH or PAS domain, respectively, should render the protein resistant to degradation. Hep3B cells were transfected with an expression vector encoding HIF-1α that was either full-length (FL) or lacked residues 33–71 of the bHLH domain (ΔbHLH), encompassing site 1, or residues 85–298 of the PAS domain (ΔPAS), encompassing site 3 (Fig. 3 N). SS1.21 induced the degradation of HIF-1α(FL) and HIF-1α(ΔPAS), but not HIF-1α(ΔbHLH), whereas SS3.2 induced the degradation of HIF-1α(FL) and HIF-1α(ΔbHLH), but not HIF-1α(ΔPAS) (Fig. 3 O). The data indicate that SS1.21 and SS3.2 bind to the HIF-1/2α bHLH and PAS domains, respectively, which blocks dimerization with HIF-1β and causes degradation of HIF-α subunits in a hydroxylation-independent and ubiquitin/proteasome-dependent manner.

**Figure S2.**
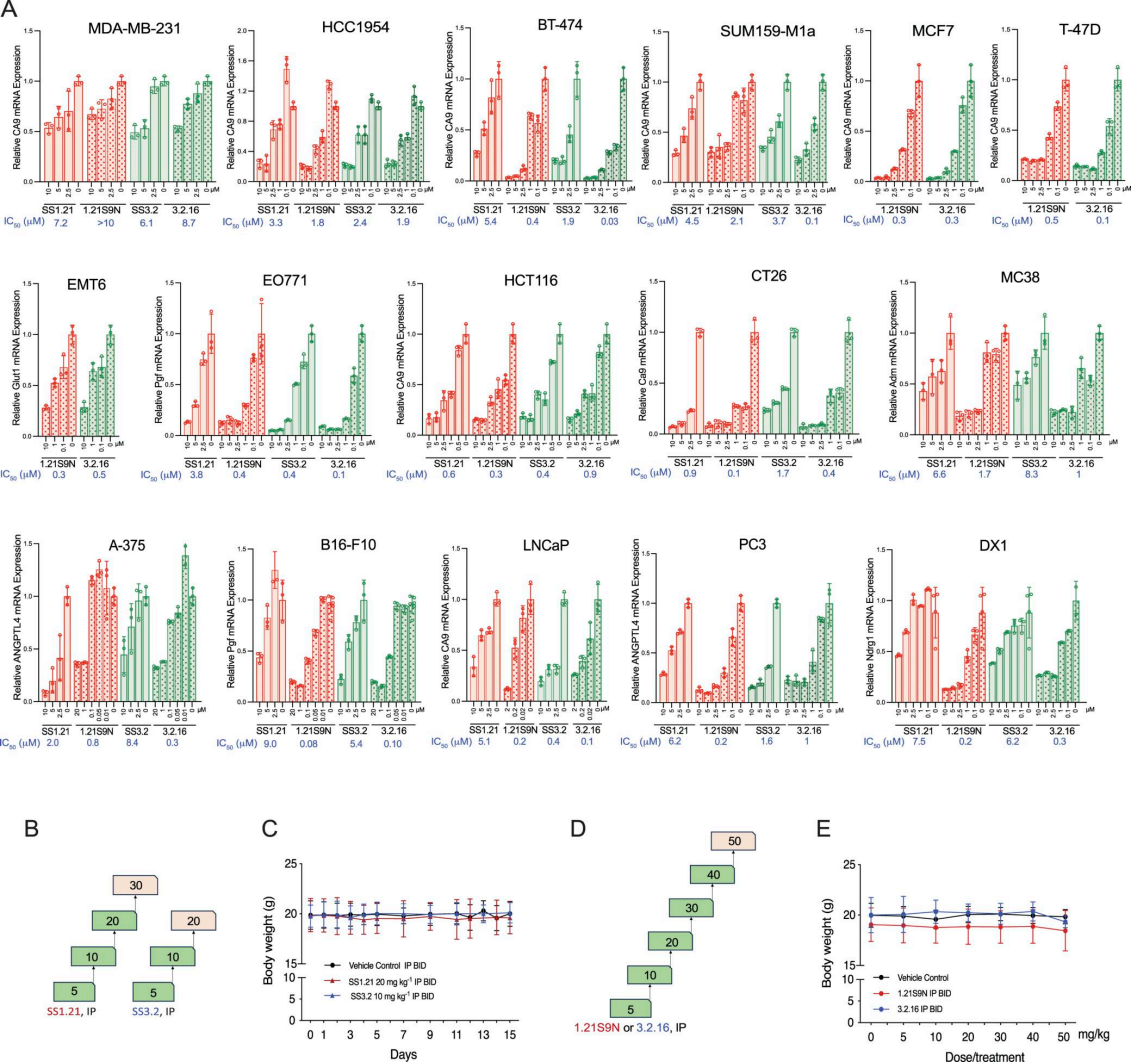
**HIFi concentration–dependent in vitro and dose-dependent in vivo effects. (A)** To calculate IC_50_ values shown in Fig. 3 P, cancer cell lines were exposed to vehicle or HIFi at the indicated concentrations for 24 h at 1% O_2_, and expression of mRNA encoding the indicated HIF target gene mRNA was analyzed by RT-qPCR (mean + SD, *n* = 3). **(B)** Dose escalation study of HIFi SS1.21 or SS3.2 was performed. Two IP doses (indicated in mg/kg) 12 h apart were administered (*n* = 3 mice each). Doses marked in green were well tolerated, whereas doses marked in pink led to decreased physical activity and/or unsteady gait in the first hour after administration. **(C)** Mice were administered vehicle or HIFi for 15 days as indicated, and BW was measured (mean + SD, *n* = 3) every 2–3 days. **(D)** Dose escalation study of HIFi 1.21S9N or 3.2.16 was performed. Two IP doses (indicated in mg/kg) 12 h apart were administered (*n* = 3 mice each). Doses marked in green were well tolerated, whereas doses marked in pink led to decreased physical activity and/or unsteady gait in the first hour after administration. **(E)** BW was measured after the second dose (mean + SD, *n* = 3).

Next, we analyzed the effect of each HIFi on hypoxia-induced HIF target gene expression in human and mouse cell lines, derived from BrCa, colorectal carcinoma (CRC), head/neck squamous cell carcinoma (HNSCC) and melanoma, and hepatocellular, lung, ovarian, pancreatic, and prostate cancer, at concentrations from 0.01 to 10 μM so that IC_50_ values could be determined (Fig. 3 P and Fig. S2 A). Among these 40 cancer cell lines, 39 were sensitive to at least one HIFi with an IC_50_ of <10 μM, and 32 cell lines were sensitive to at least one HIFi with an IC_50_ of <1 μM. Thus, these compounds show broad and potent HIFi activity in vitro.

### SS1.21 and SS3.2 inhibit CRC, HNSCC, and BrCa tumor growth

**Figure 4.**
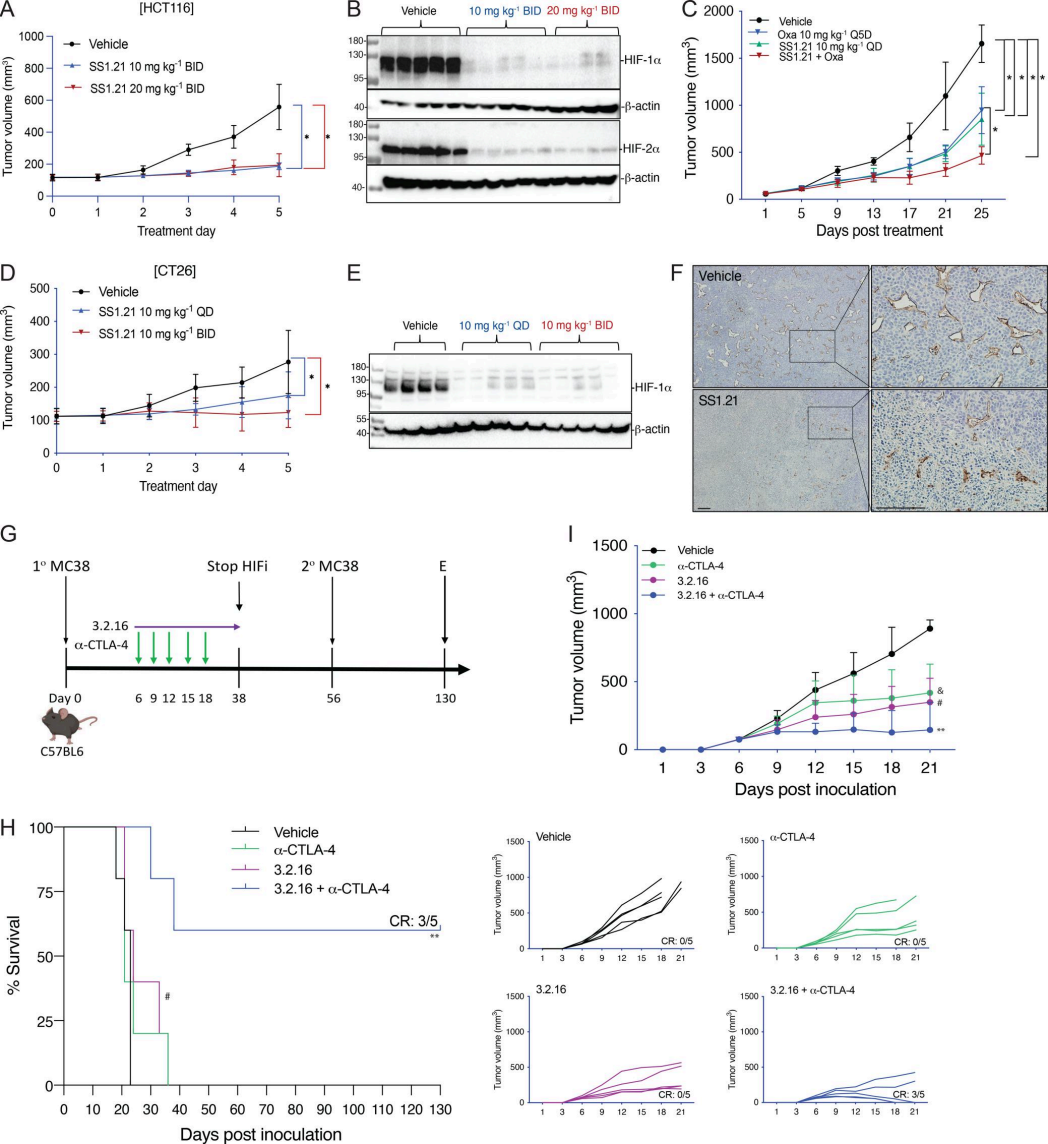
**SS1.21 and 3.2.16 inhibit colorectal cancer growth. (A)** Nude mice bearing 150-mm^3^ HCT116 xenografts were treated with vehicle or SS1.21, and tumor growth was monitored (mean + SD, *n* = 5); *P < 0.05 by two-way ANOVA. **(B)** Tumors were harvested 4 h after the last dose, and immunoblot assays were performed. **(C)** Mice were injected subcutaneously with HCT116 cells and, when a tumor was palpable, were treated with vehicle, Oxa, SS1.21, or Oxa + SS1.21, and tumor volumes were determined (mean + SD, *n* = 5); *P < 0.05 by two-way ANOVA. **(D)** Balb/c mice bearing 100-mm^3^ CT26 tumors were treated with vehicle or SS1.21, and tumor volumes were determined (mean + SD, *n* = 5); *P < 0.05 by two-way ANOVA with the Bonferroni post-test. **(E)** Tumors were harvested 4 h after the last dose, and immunoblot assays were performed. **(F)** Tumor sections from mice treated with vehicle or SS1.21 were analyzed by immunohistochemistry using anti-CD31 antibody. **(G)** C57BL/6 mice were injected with MC38 cells and treated with vehicle, α-CTLA-4 antibody (200 μg Q3D), 3.2.16 (40 mg/kg BID), or both. **(H)** Kaplan–Meier analysis of mouse survival is shown. **(I)** Mean (top panel) and individual (bottom panels) tumor growth are presented. ^…^P < 0.0001 versus vehicle, ^#^P < 0.0001 versus vehicle, **P < 0.0001 versus α-CTLA-4 by two-way ANOVA with Tukey’s post-test. The number of mice with a CR is shown. Scale bar, 500 µm. Oxa, oxaliplatin. Source data are available for this figure: SourceData F4.

**Figure S3.**
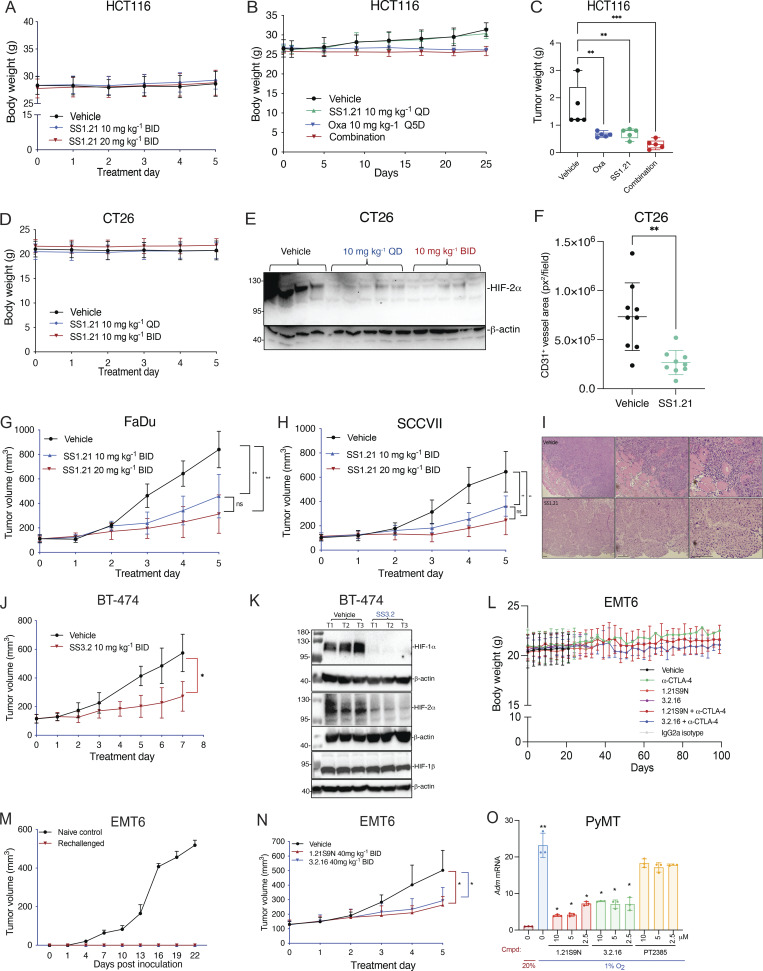
**CRC, HNSCC, and BrCa tumor models.** Related to Figs. 4 and 5. **(A–C)** Additional data from the HCT116 tumor model, including mouse BW (A and B) and final tumor weight (C), are shown. Data are presented as the mean ± SD (*n* = 5) and analyzed by one-way ANOVA with the Sidak multiple comparisons. **P < 0.01; ***P < 0.001. **(D–F)** Additional data from the CT26 model (presented in Fig. 4. D–F) are shown including: mouse BW (D); HIF-2α immunoblot assay of tumor lysates (E); and CD31^+^ vessel area (square pixels per field) in tumor sections from mice treated with vehicle or SS1.21 (10 mg/kg QD) (F). Data are presented as the mean ± SD. **P < 0.01 by Student’s t test. **(G–I)** HNSCC tumor models were studied: FaDu human HNSCC xenografts in nude mice (G) and syngeneic SCC-VII tumors in C3H mice (H). Mice were treated with vehicle or SS1.21 (10 or 20 mg/kg BID IP × 5 days). Tumor volumes are presented as the mean ± SD (*n* = 5). **P < 0.01, two-way ANOVA with Tukey’s post-test; ns, not significant. Representative hematoxylin-and-eosin–stained FaDu tumor sections from mice treated with vehicle or SS1.21 (20 mg/kg) are shown illustrating resection margins (I). **(J)** Mice bearing BT-474 human orthotopic breast tumors were treated with vehicle or SS3.2 (10 mg/kg BID IP × 7 days). Data are presented as the mean ± SD (*n* = 5). *P < 0.05 by two-way ANOVA. **(K)** BT-474 tumor lysates were subjected to immunoblot assays using antibodies for HIF-1α, HIF-2α, HIF-1β, and β-actin. **(L–N)** Additional data from the EMT6 model, including mouse BW (L); results of rechallenge experiment in which control naïve mice or previously treated, tumor-free mice received an injection of tumor cells (M); and tumor growth in mice treated with vehicle, 1.21S9N (40 mg/kg BID), or 3.2.16 (40 mg/kg BID) with data presented as the mean ± SD (*n* = 5); *P <0.05 by two-way ANOVA with Tukey’s post-test (N). RNA and protein extracted from these tumors were analyzed in Fig. 5, I and J, respectively. **(O)** Primary cancer cells from *MMTV-PyMT* mammary tumors were treated with vehicle or the indicated HIFi at 20% or 1% O_2_ for 24 h, and Adm mRNA expression was analyzed by RT-qPCR. Data are presented as the mean ± SD (*n* = 3). *P < 0.05, **P < 0.01 by two-way ANOVA with Dunnett’s post-test. Scale bar, 500 µm. Source data are available for this figure: SourceData FS3.

Next, we performed a dose escalation study in vivo. Mice tolerated up to 20 mg/kg of SS1.21 or 10 mg/kg of SS3.2 by intraperitoneal (IP) injection with no change in activity, whereas mice were noticeably less active for ∼1 h after administration of higher doses (Fig. S2 B). Mice tolerated administration of SS1.21 or SS3.2 twice daily (BID) for 15 days with no change in body weight (BW) (Fig. S2 C). Based on these results, athymic nude mice were given a subcutaneous injection of HCT116 human CRC cells, and when tumors reached a volume of 150 mm^3^, the mice were administered SS1.21, either 10 or 20 mg/kg BID, for 5 days. Compared with vehicle, SS1.21 significantly inhibited tumor growth (Fig. 4 A). There was no effect of SS1.21 on mouse appearance, behavior, or BW (Fig. S3 A). Immunoblot assays revealed strong expression of HIF-1α and HIF-2α in tumors from vehicle-treated mice but not in tumors from SS1.21-treated mice (Fig. 4 B).

Next, we administered SS1.21 or oxaliplatin, starting when tumors became palpable. Whereas treatment with oxaliplatin, which is a cytotoxic chemotherapy used to treat CRC patients, negatively affected mouse BW, SS1.21 had no detrimental effect when administered as monotherapy (Fig. S3 B). SS1.21 was as effective as oxaliplatin in decreasing tumor growth (Fig. 4 C) and final tumor weight (Fig. S3 C), and the combination inhibited tumor growth more than either drug alone (Fig. 4 C), with no increased toxicity as compared to oxaliplatin alone (Fig. S3 B).

To investigate the effect of SS1.21 in immunocompetent mice, CT26 mouse CRC cells were injected subcutaneously into syngeneic Balb/c mice, and when tumors reached 100 mm^3^, the mice were treated with vehicle or SS1.21, either once daily (QD) or BID. SS1.21 inhibited tumor growth (Fig. 4 D) but had no effect on mouse appearance, behavior, or BW (Fig. S3 D). Immunoblot assays revealed high HIF-1α and HIF-2α expression in tumors from vehicle-treated mice but not from SS1.21-treated mice (Fig. 4 E and Fig. S3 E). The luminal area of tumor blood vessels was significantly decreased in SS1.21-treated mice (Fig. 4 F and Fig. S3 F), indicating that the HIFi potently impaired tumor vascularization.

To investigate the sensitivity of HNSCC to SS1.21, human FaDu or mouse SCC-VII cells were injected subcutaneously into nude or syngeneic C3H mice, respectively. SS1.21 administration for 5 days inhibited growth of FaDu and SCC-VII tumors (Fig. S3, G and H). FaDu tumors from vehicle-treated mice were difficult to resect due to extensive infiltration of surrounding skeletal muscle by cancer cells, whereas tumors from SS1.21-treated mice were easily resected with clean margins (Fig. S3 I), indicating a less invasive phenotype.

Next, we evaluated the activity of SS3.2 in vivo by performing orthotopic implantation of BT-474 human BrCa cells into the mammary fat pad (MFP) of female nude mice. When tumor volume reached 150 mm^3^, the mice were treated with SS3.2, which inhibited tumor growth (Fig. S3 J) and induced HIF-1α and HIF-2α degradation (Fig. S3 K). The data presented in Fig. 4, A–F; and Fig. S3, A–K indicate that SS1.21 and SS3.2 induce HIF-1/2α degradation and decrease tumor growth in immunodeficient and immunocompetent mouse models of CRC, HNSCC, and BrCa.

### 3.2.16 enhances the antitumor activity of anti-CTLA-4 antibody in CRC

We developed 3.2.16 as a more potent derivative of SS3.2 (Fig. 1 E and Fig. 3 P). In addition, 3.2.16 was better tolerated and could be administered at a higher dose of 40 mg/kg IP (Fig. S2, D and E). The nonspecific HIFi echinomycin was reported to potentiate the effect of α-CTLA-4 antibody in the MC38 CRC model (Bailey et al., 2022). We injected MC38 cells subcutaneously into syngeneic C57BL/6 mice. Once tumors were palpable, mice were treated by IP injection of vehicle, α-CTLA-4 (200 μg Q3D × 5 doses), 3.2.16 (40 mg/kg BID), or both (Fig. 4 G). Tumors grew rapidly in vehicle-treated mice, which required euthanasia by days 18–23 (Fig. 4, H and I). Tumor growth in mice treated with α-CTLA-4 or 3.2.16 alone was significantly decreased (Fig. 4 I), but mice required euthanasia by day 36 (Fig. 4 H). In contrast, three out of five mice treated with α-CTLA-4 + 3.2.16 had a complete response (CR) with no palpable tumor after day 21 (Fig. 4, H and I). These mice remained tumor-free despite discontinuation of 3.2.16 on day 38. On day 56, these mice received a second injection of MC38 cells, and no tumor recurrence was observed as of day 130, when the study was terminated (Fig. 4, G and H).

### HIFi therapy overcomes resistance to α-CTLA-4 in a BrCa model

**Figure 5.**
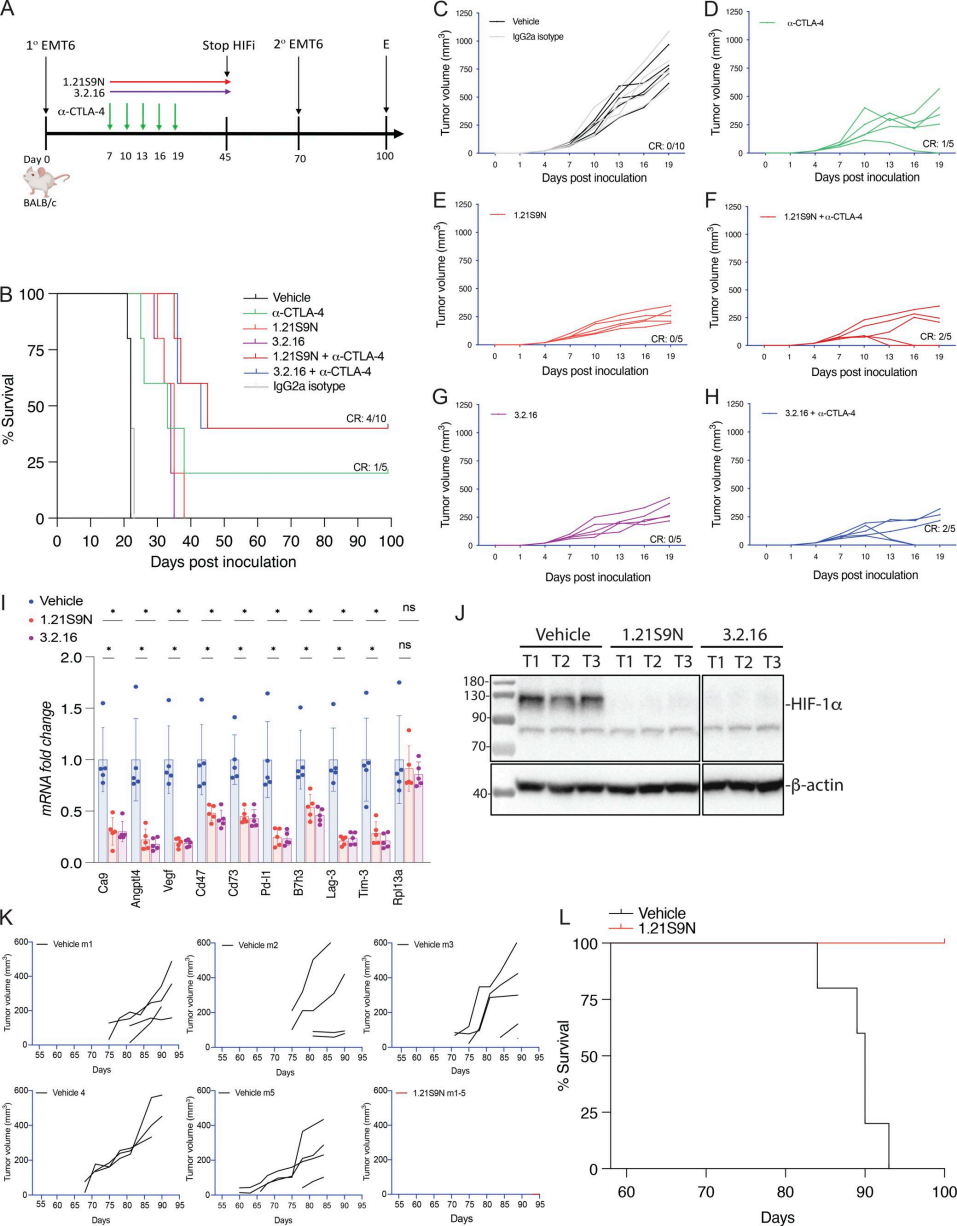
**Effect of HIFi therapy in BrCa models. (A)** EMT6 cells were injected into the MFP of Balb/c mice, which were treated by IP injection of: vehicle or IgG2a isotype control antibody (200 μg Q3D); α-CTLA-4 (200 μg Q3D); 1.21S9N or 3.2.16 (40 mg/kg BID); or α-CTLA-4 + HIFi. **(B)** Kaplan–Meier analysis of mouse survival is shown. **(C-H)** For each treatment group, individual tumor growth curves are shown. The number of mice in each group that achieved a durable CR on day 100 (after tumor rechallenge on day 70) is indicated. **(I and J)** Mice with 150-mm^3^ EMT6 tumors in the MFP were treated with vehicle or either 1.21S9N or 3.2.16 (40 mg/kg BID) for 5 days, tumors were harvested 4 h after the last dose, and mRNA expression (I) and protein expression (J) were analyzed by RT-qPCR (mean + SD, *n* = 5; *P < 0.05 by one-way ANOVA with the Bonferroni post-test) and immunoblot assays, respectively. **(K and L)** Tumor growth curves for individual mice (K) and survival (L) of *MMTV-PyMT* mice treated by OG administration of either vehicle or 1.21S9N (60 mg/kg BID) for 6 wk starting on day 53 of life (*n* = 5 mice per group). Source data are available for this figure: SourceData F5.

EMT6 triple-negative BrCa (TNBC) cells are resistant to α-CTLA-4 treatment (Khononov et al., 2021; Samanta et al., 2020). 1.21S9N (Fig. 1 E) was better tolerated than SS1.21 (Fig. S2, B–E) and, like 3.2.16, could be administered IP at 40 mg/kg BID. We compared the therapeutic effect of combining α-CTLA-4 with either 1.21S9N or 3.2.16. EMT6 cells were injected into the MFP of syngeneic Balb/c mice, and treatment was initiated when tumors became palpable on day 7 (Fig. 5 A). Compared with control mice, monotherapy significantly increased survival time and decreased tumor growth, but the greatest effect was observed with combination therapy (α-CTLA-4 + 1.21S9N or 3.2.16), which led to CR in 4/10 mice, as compared to 1/5 mice treated with α-CTLA-4 alone (Fig. 5, B–H). There was no significant effect of treatment on BW (Fig. S3 L). When tumor-free mice were rechallenged with EMT6 cells injected into the adjacent MFP on day 70 (Fig. 5 A), none of the mice developed tumors, in contrast to injection of naïve control mice, which all developed tumors (Fig. S3 M). Analysis of RNA from tumors of mice administered HIFi monotherapy for 5 days (Fig. S3 N) revealed decreased expression of markers of angiogenesis (Angptl4, Vegf), T cell exhaustion (Lag3, Tim3), immune checkpoint proteins (B7h3, Pdl1), and other mediators of immunosuppression (Ca9, Cd47, Cd73), whereas Rpl13a expression was unchanged (Fig. 5 I). HIF-1α levels were markedly decreased in tumors from HIFi-treated as compared to vehicle-treated mice (Fig. 5 J).

To further assess HIFi efficacy, we utilized the *MMTV-PyMT* genetically engineered mouse model, which is widely employed as a preclinical model for TNBC. In this autochthonous model, the expression of the polyoma virus middle T antigen (PyMT) driven by the mouse mammary tumor virus promoter leads to the development of palpable tumors starting around day 53 (Attalla et al., 2021; Guy et al., 1992). To evaluate the efficacy of HIFi therapy, cancer cells were isolated from mammary carcinomas of *MMTV-PyMT* female mice (Sun et al., 2022) and incubated at 20% or 1% O_2_ for 24 h in the presence of vehicle, 1.21S9N, 3.2.16, or PT2385. Treatment with 1.21S9N or 3.2.16 inhibited hypoxia-induced adrenomedullin (Adm) mRNA expression, whereas PT2385 had no significant effect (Fig. S3 O). Orogastric (OG) administration of vehicle or 1.21S9N BID to *MMTV-PyMT* female mice was initiated on day 53, and tumor growth was monitored. All five vehicle-treated mice developed palpable tumors between days 60 and 75 and required euthanasia by day 94, whereas none of the five mice treated with 1.21S9N developed palpable tumors during the treatment period (Fig. 5, K and L).

### HIFi enhances response to α-CTLA-4 in melanoma and prostate cancer models

**Figure 6.**
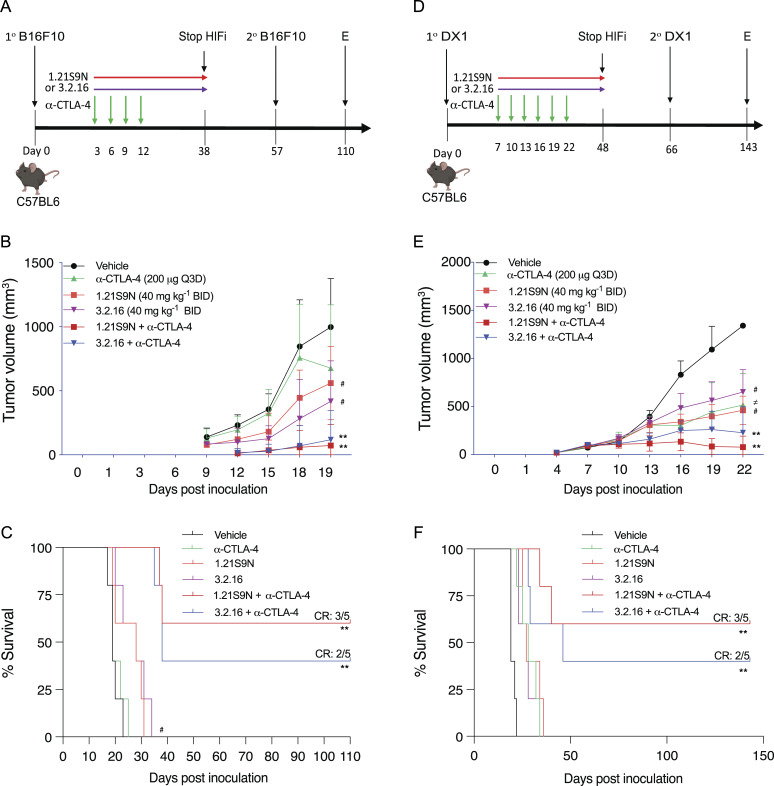
**Effect of HIFi therapy in melanoma and prostate cancer models. (A)** C57BL/6 mice were injected subcutaneously with B16F10 melanoma cells and treated with vehicle, HIFi (1.21S9N or 3.2.16), α-CTLA-4, or HIFi + α-CTLA-4. **(B)** Mean tumor volumes are shown (mean + SD, *n* = 5). ^#^P < 0.05 versus vehicle, **P < 0.005 versus α-CTLA-4 by two-way ANOVA with Tukey’s post-test. **(C)** Kaplan–Meier survival curves are shown. **(D)** C57BL/6 mice were injected with DX1 prostate cancer cells and treated with vehicle, HIFi, α-CTLA-4, or HIFi + α-CTLA-4. **(E)** Mean tumor volumes are shown (mean + SD, *n* = 5). ^≠^P < 0.05 versus vehicle, ^#^P <0.05 versus vehicle, **P < 0.05 versus α-CTLA-4 by two-way ANOVA with Tukey’s post-test. **(F)** Kaplan–Meier survival curves are shown.

**Figure S4.**
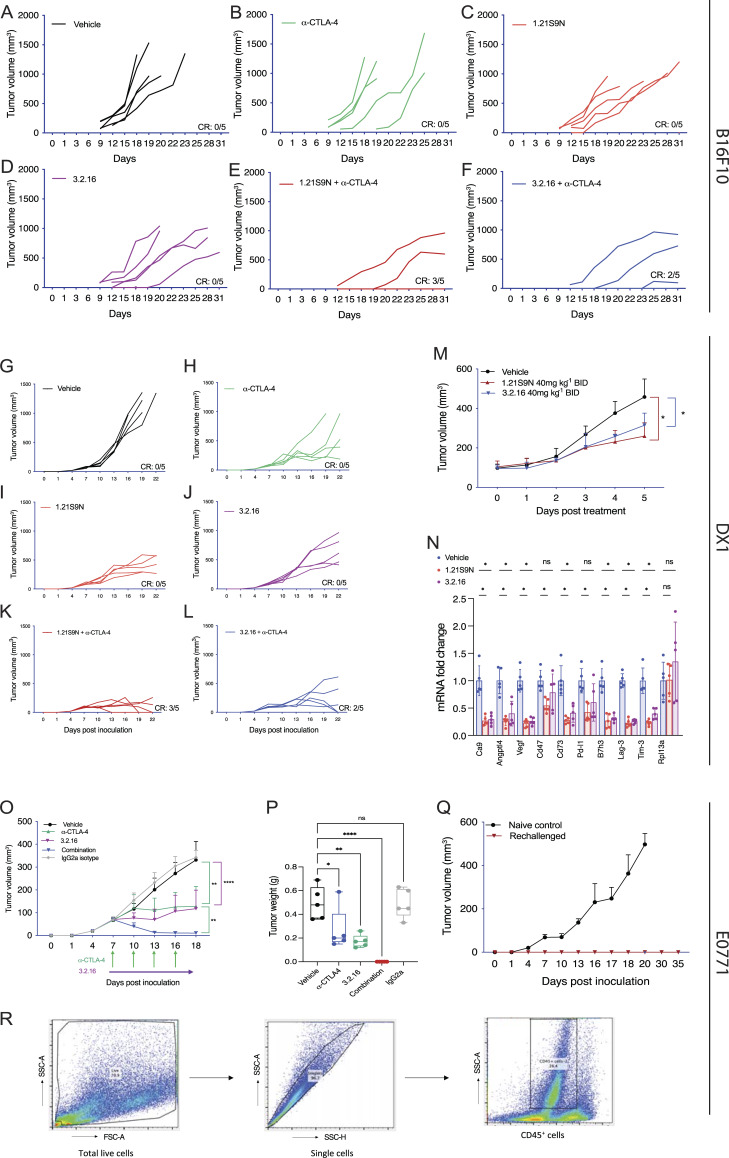
**Melanoma, prostate, and breast tumor models.** Related to Figs. 6 and 7. **(A–L)** B16F10 melanoma (A–F) and DX1 prostate cancer (G–L) individual tumor growth curves under indicated treatments are shown. **(M)** Mice bearing DX1 tumors were treated with vehicle or HIFi (40 mg/kg BID IP) for 5 days. Tumor volumes are presented as the mean ± SD (*n* = 5); *P < 0.05 versus vehicle by two-way ANOVA with Tukey’s post-test. **(N)** RNA was isolated from DX1 tumors 4 h after the last treatment and analyzed by RT-qPCR (mean + SD, *n* = 5). *P < 0.05 by one-way ANOVA with the Bonferroni post-test. **(O)** E0771 tumor volume measurements are shown (mean + SD, *n* = 5). **P < 0.01 by two-way ANOVA with Tukey’s post-test. **(P)** E0771 final tumor weights are presented as the mean ± SD, *n* = 5. **P < 0.01; ****P < 0.0001; ns by one-way ANOVA with Dunnett’s post-test. **(Q)** E0771 tumor volumes (mean ± SD) in rechallenged versus naïve mice are shown. **(R)** Gating strategy for the flow cytometry data presented in Fig. 7, G–L is shown.

B16F10 is a highly aggressive model of immunotherapy-resistant melanoma (Sharma et al., 2019). Pharmacologic or genetic inhibition of HIF activity in B16F10 cells led to improved responses to treatment with a peptide vaccine and α-PD-1 (Lequeux et al., 2021). We treated syngeneic C57BL/6 mice bearing subcutaneous B16F10 tumors with HIFi, α-CTLA-4, or both (Fig. 6 A). Although monotherapy delayed tumor growth, combination therapy was synergistic (Fig. 6 B), with tumor eradication in three of five mice treated with 1.21S9N and two of five mice treated with 3.2.16 (Fig. S4, A–F), and even after a second injection of B16F10 cells on day 57, these mice remained tumor-free through day 110, when the study was terminated (Fig. 6, A and C).

DX1 prostate cancer cells are resistant to combination therapy with androgen receptor inhibitor enzalutamide and α-PD-1 or α-CTLA-4, which was attributed to the expression of another immune checkpoint receptor, B7-H3 (Shi et al., 2023; Zhao et al., 2020). We injected DX1 cells subcutaneously into syngeneic C57BL/6 male mice, which were treated with α-CTLA-4, HIFi, or both; androgen receptor inhibitor was not administered (Fig. 6 D). Compared with vehicle, monotherapy significantly inhibited tumor growth but no CR rate was achieved (Fig. 6, E and F; and Fig. S4, G–J). Treatment with HIFi increased the sensitivity of DX1 tumors to α-CTLA-4, leading to CR in five of 10 mice (Fig. 6, E and F; and Fig. S4, K and L). Rechallenge with DX1 cells after discontinuation of HIFi therapy (Fig. 6 D) did not result in tumor growth.

We also treated 150-mm^3^ DX1 prostate tumors with vehicle or HIFi for 5 days (Fig. S4 M) and analyzed mRNA expression 4 h after the last dose. The expression of angiogenic factors (Angptl4, Vegf), immune checkpoint proteins (B7h3, Pd-l1), markers of T cell exhaustion (Lag-3, Tim-3), and mediators of immune evasion (Ca9, Cd47, Cd73, Vegf) was significantly decreased in tumors from mice treated with HIFi (Fig. S4 N).

### HIFi therapy alters the TIME

**Figure 7.**
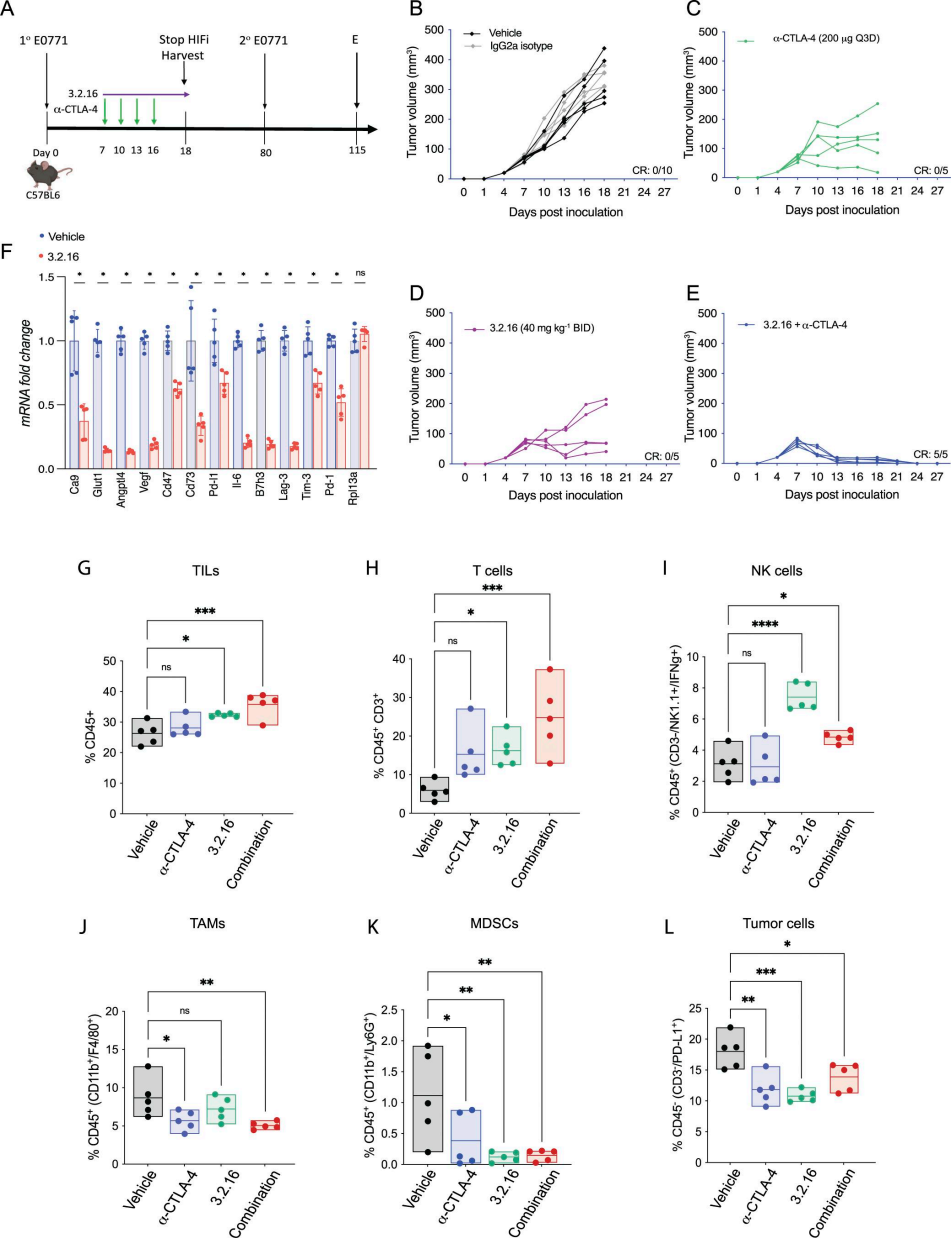
**Effect of treatment with 3.2.16 in the E0771 BrCa model. (A)** C57BL/6 mice received an injection of E0771 cells into the MFP and were treated with vehicle or IgG2a, α-CTLA-4, 3.2.16, or α-CTLA-4 + 3.2.16. **(B–E)** For each treatment group, individual tumor growth curves are presented. The number of mice in each group that achieved a CR on day 27 is indicated. **(F)** Tumors from mice treated with vehicle or 3.2.16 were analyzed for mRNA expression, shown as the mean + SD (*n* = 5). *P < 0.05 by Student’s *t* test. **(G-L)** Mice with 150-mm^3^ E0771 tumors in the MFP were treated with vehicle, α-CTLA-4, 3.2.16, or α-CTLA-4 + 3.2.16 (combination) and analyzed by flow cytometry. The percentage of CD45^+^ TILs (G), CD45^+^CD3^+^ T cells (H), CD45^+^CD3^-^NK1.1^+^ IFNγ^+^ NK cells (I), CD45^+^CD11b^+^ F4/80^+^ TAMs (J), CD45^+^CD11b^+^Ly6G^+^ MDSCs (K), and CD45^−^CD3^-^PDL1^+^ tumor cells (L) were determined. Data are presented as individual values and mean (*n* = 5). *P < 0.05; **P < 0.01; ***P < 0.001; ****P < 0.0001 by one-way ANOVA with Dunnett’s post-test. TILs, tumor-infiltrating leukocytes.

We implanted E0771 BrCa cells into the MFP of syngeneic C57BL/6 female mice. When tumors became palpable on day 7, we started treatment with 3.2.16, α-CTLA-4, or both (Fig. 7 A). Treatment with 3.2.16 or α-CTLA-4 suppressed tumor growth in all mice initially, but two of five tumors in 3.2.16-treated mice and four of five tumors in α-CTLA-4-treated mice eventually escaped suppression (Fig. 7, B–D). In contrast, combination therapy resulted in tumor rejection in five of five mice (Fig. 7 E and Fig. S4 O). HIFi treatment was terminated on day 18, and tumors were harvested and weighed (Fig. S4 P). On day 80, mice that remained tumor-free were rechallenged with a second injection of E0771 cells into the adjacent MFP (Fig. 7 A). Naïve animals injected with E0771 cells formed tumors rapidly, whereas none of the mice previously treated with 3.2.16 + α-CTLA-4 developed a tumor after rechallenge (Fig. S4 Q). Analysis of E0771 tumors from mice treated with 3.2.16 revealed decreased mRNA expression of markers of angiogenesis (Angptl4, Vegf), T cell exhaustion (Lag-3, Pd-1, Tim-3), and other mediators of immunosuppression (Ca9, Cd47, Cd73, Glut1, Il-6, Pd-l1, Vegf), whereas Rpl13a expression was unchanged (Fig. 7 F).

The mRNA data from EMT6 and E0771 tumors indicate that treatment of BrCa with HIFi enhances ICB by disrupting multiple mechanisms of immune evasion leading to alterations in the TIME. To test this hypothesis, mice bearing 150-mm^3^ E0771 tumors were treated with vehicle, α-CTLA-4, 3.2.16, or both, and tumors were harvested for analysis by flow cytometry (Fig. S4 R). The percentages of total CD45^+^ tumor-infiltrating leukocytes (Fig. 7 G), CD45^+^CD3^+^ T cells (Fig. 7 H), and CD45^+^CD3^-^NK1.1^+^IFNγ^+^ cytolytic NK cells (Fig. 7 I) were all increased by treatment with 3.2.16, either alone or in combination with α-CTLA-4. In contrast, the percentages of CD45^+^CD11b^+^F4/80^+^ tumor-associated macrophages (TAMs) (Fig. 7 J) and CD45^+^CD11b^+^Ly6G^+^ myeloid-derived suppressor cells (MDSCs) (Fig. 7 K) were decreased in tumors from mice treated with 3.2.16 + α-CTLA-4. The percentage of CD45^−^CD3^−^PDL1^+^ cancer cells was decreased in all three treatment groups (Fig. 7 L). These data indicate that 3.2.16 + α-CTLA-4 converted an immunosuppressive TIME, dominated by MDSCs and TAMs, into one favoring antitumor immunity, populated by NK and T cells.

### Effect of HIF inhibitors on response to immunotherapy: A meta-analysis

**Figure 8.**
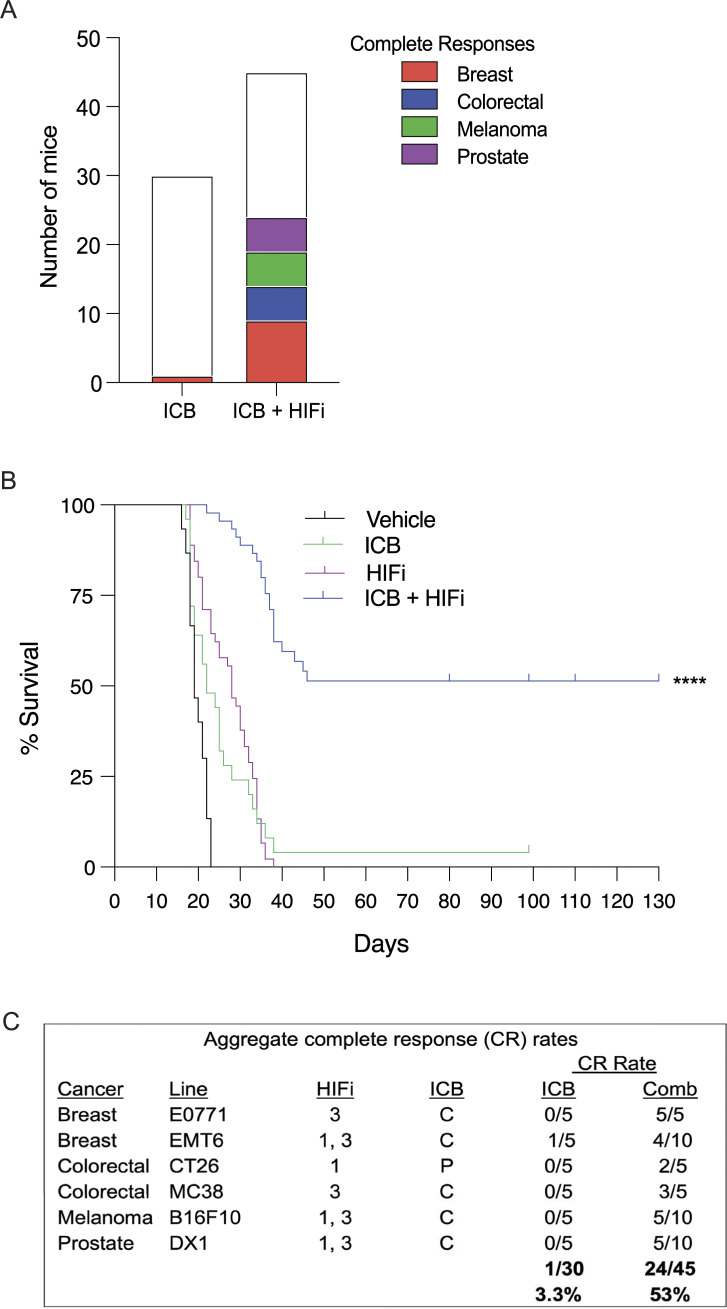
**CR and survival analysis. (A)** Aggregate CR in mice with transplanted tumors treated with ICB or with ICB + HIFi is presented. **(B)** Aggregate Kaplan–Meier survival curves for mice treated with vehicle, ICB, HIFi, or ICB + HIFi. ****P < 0.0001 versus ICB by the log-rank test. **(C)** Aggregate CR rates. Mice were treated with ICB alone (α-CTLA-4 [C] or α-PD-1 [P]) or in combination (Comb) with HIFi (1.21S9N [1] or 3.2.16 [3]) across six mouse cancer models.

Our experimental strategy was to survey the effect of HIFi therapy on tumor growth and the response to ICB in syngeneic mouse models of melanoma, BrCa, CRC, and prostate cancer. Because the number of mice in each individual treatment group was small, we performed a meta-analysis combining the results of all cancer types and treatment protocols. The aggregate CR to ICB monotherapy was 1/30 (3.3%), as compared to 24/45 (53%) in mice treated with ICB + HIFi (Fig. 8).

### Analysis of HIFi pharmacodynamics and pharmacokinetics

**Figure S5.**
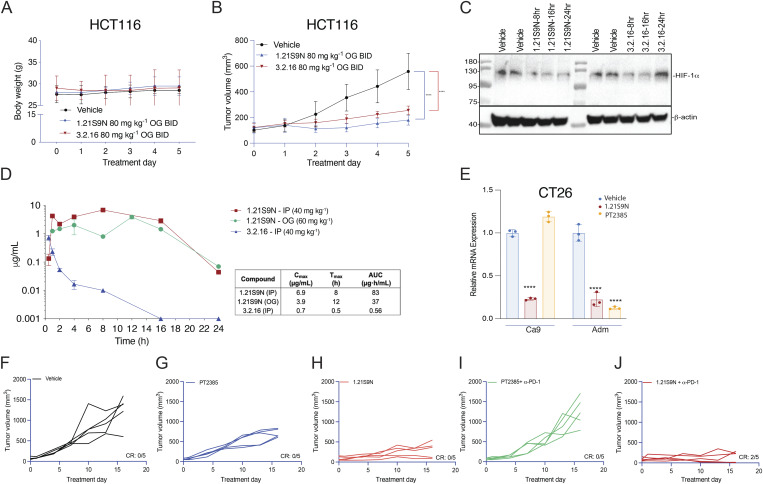
**Analysis of mice treated with 1.21S9N or 3.2.16.** Related to Fig. 8. (**A and B)** Nude mice bearing HCT116 tumors were administered vehicle or HIFi (80 mg/kg OG BID). BW (A) and tumor volume (B) were measured. Data are presented as the mean ± SD, *n* = 5. ***P < 0.001 versus vehicle by two-way ANOVA with Dunnett’s multiple comparisons. **(C)** Lysates were prepared from tumors collected 8, 16, or 24 h after the last dose of HIFi or vehicle and probed for HIF-1α and β-actin expression by immunoblot assay. **(D)** Plasma concentration of HIFi (mean ± SD, *n* = 1-3 mice each) was measured at the indicated time (hours) following IP or OG administration of a single dose. **(E)** CT26 cells were incubated for 24 h at 20% O_2_ with vehicle or at 1% O_2_ with vehicle, 1.21S9N, or PT2385. RNA was isolated and analyzed by RT-qPCR using primers specific for Ca9 and Adm. Data are presented as the mean ± SD (*n* = 3). ****P < 0.0001 versus vehicle–1% O_2_, by two-way ANOVA with Dunnett’s multiple comparisons. **(F–J)** Individual CT26 tumor growth curves are shown for data presented in Fig. 9 A. Source data are available for this figure: SourceData FS5.

HCT116 tumor-bearing mice tolerated OG administration of HIFi at 80 mg/kg BID OG for 5 days without adverse effects on appearance, behavior, or BW and blocked HCT116 tumor growth (Fig. S5, A and B). Intratumoral HIF-1α levels were inhibited for 16 and 24 h after the last dose of 3.2.16 and 1.21S9N, respectively (Fig. S5 C). Pharmacokinetic profiling (Fig. S5 D) supported these pharmacodynamic data. Following a single dose of 40 mg/kg IP, 1.21S9N achieved a plasma C_max_ = 6.9 µg/ml at T_max_ = 8 h and AUC_0–24_ = 83 µg·h/ml, whereas 3.2.16 showed lower and shorter plasma exposure with C_max_ = 0.7 µg/ml at T_max_ = 0.5 h and AUC_0–24_ = 0.56 µg·h/ml). After 60 mg/kg OG, 1.21S9N produced systemic exposure with C_max_ = 3.9 µg/ml at 12 h and AUC_0–24_ = 37 µg·h/ml (Fig. S5 D).

### Efficacy and safety of dual HIF-1/2 versus selective HIF-2 inhibition

**Figure 9.**
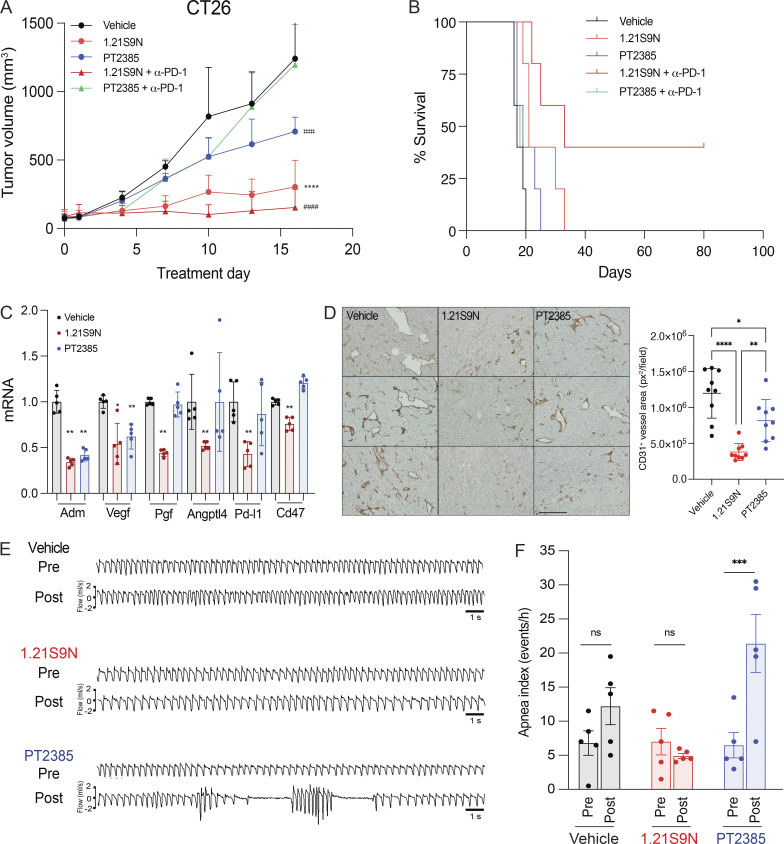
**Effect of dual HIF-1/2 inhibition compared to selective HIF-2 inhibition. (A)** Balb/c mice bearing CT26 tumors were treated by OG administration of vehicle, PT2385 (60 mg/kg QD), 1.21S9N (60 mg/kg QD) or by IP injection of α-PD-1 antibody (200 mg Q3D) in combination with either PT2385 or 1.21S9N, and tumor volumes were determined (mean + SD, *n* = 5). ^‡‡‡‡^P < 0.0001 for PT2385 vs vehicle; ****P < 0.0001 for 1.21S9N vs vehicle or PT2385; ^####^P < 0.0001 for 1.21S9N + α-PD-1 vs PT2385 + α-PD-1 by two-way ANOVA with Tukey's post-test. **(B)** Kaplan-Meier analysis of survival is shown. **(C)** Tumors were harvested 4 hours after the last dose for analysis of mRNA expression. Results are presented as mean ± SD (*n* = 5). *P < 0.05; **P < 0.01 vs vehicle; one-way ANOVA with Bonferroni post-test. **(D)** Tumor sections were analyzed by immunohistochemistry using anti-CD31 antibody. Results are presented as mean ± SD (*n* = 3). *P < 0.05; **P < 0.01 vs vehicle; one-way ANOVA with Sidak post-test. Scale bar, 500 µm. **(E and F)** Breathing was monitored in mice pre- and post-treatment with vehicle, 1.21S9N or PT2385 (30 mg/kg BID OG x 3 doses) by whole body plethysmography and representative ventilatory tracings are shown (E). The apnea index (events per hour) is plotted for each treatment group (mean ± SEM, *n* = 5 mice per group). ***P < 0.001 vs vehicle by two-way ANOVA with Holm-Sidak post-test (F).

Treatment of CT26 CRC cells with HIF-2 inhibitor PT2385 inhibited hypoxia-induced expression of Adm but not Ca9 mRNA, whereas 1.21S9N inhibited both (Fig. S5 E). PT2385 (60 mg/kg QD OG) was active in a mouse model of RCC (Wallace et al., 2016). We administered PT2385 or 1.21S9N at this dose/schedule to CT26 tumor-bearing mice. PT2385 treatment resulted in 30% tumor growth inhibition, whereas 1.21S9N treatment led to 70% tumor growth inhibition as monotherapy (Fig. 9 A and Fig. S5, F–H). We tested whether 1.21S9N or PT2385 had a synergistic effect when administered in combination with α-PD-1 antibody. Treatment with 1.21S9N increased survival when combined with α-PD-1 therapy, with a CR rate of 40% for 1.21S9N + α-PD1 compared with 0% for PT2385 + α-PD1 (Fig. 9 B and Fig. S5, I and J). Analysis of tumor tissue demonstrated that 1.21S9N monotherapy inhibited expression of mRNAs encoding the angiogenic factors Adm, Angptl4, Pgf, and Vegf, as well as the immune checkpoint receptors Pd-l1 and Cd47, whereas PT2385 significantly inhibited Adm and Vegf only (Fig. 9 C). Tumors harvested from 1.21S9N-treated mice showed significantly decreased vascularization compared to tumors treated with vehicle or PT2385 (Fig. 9 D). Thus, dual HIF-1/2 inhibition had greater effects on gene expression, leading to enhanced suppression of tumor growth and vascularization.

In prior studies, PT2385 inhibited mouse ventilatory responses to hypoxia (Cheng et al., 2020). We treated mice with PT2385 or 1.21S9N at the same dose and schedule (30 mg/kg q12h × 3 doses). Whole-body plethysmography revealed disordered breathing with increased apnea episodes (cessation of breathing) in mice treated with PT2385 as compared to vehicle, whereas 1.21S9N had no adverse effect on breathing (Fig. 9, E and F). Thus, head-to-head testing in mice indicates superior efficacy (Fig. 9, A–D) and safety (Fig. 9, E and F) of the dual HIFi 1.21S9N compared with the HIF-2–selective inhibitor PT2385.

### Safety studies

**Figure 10.**
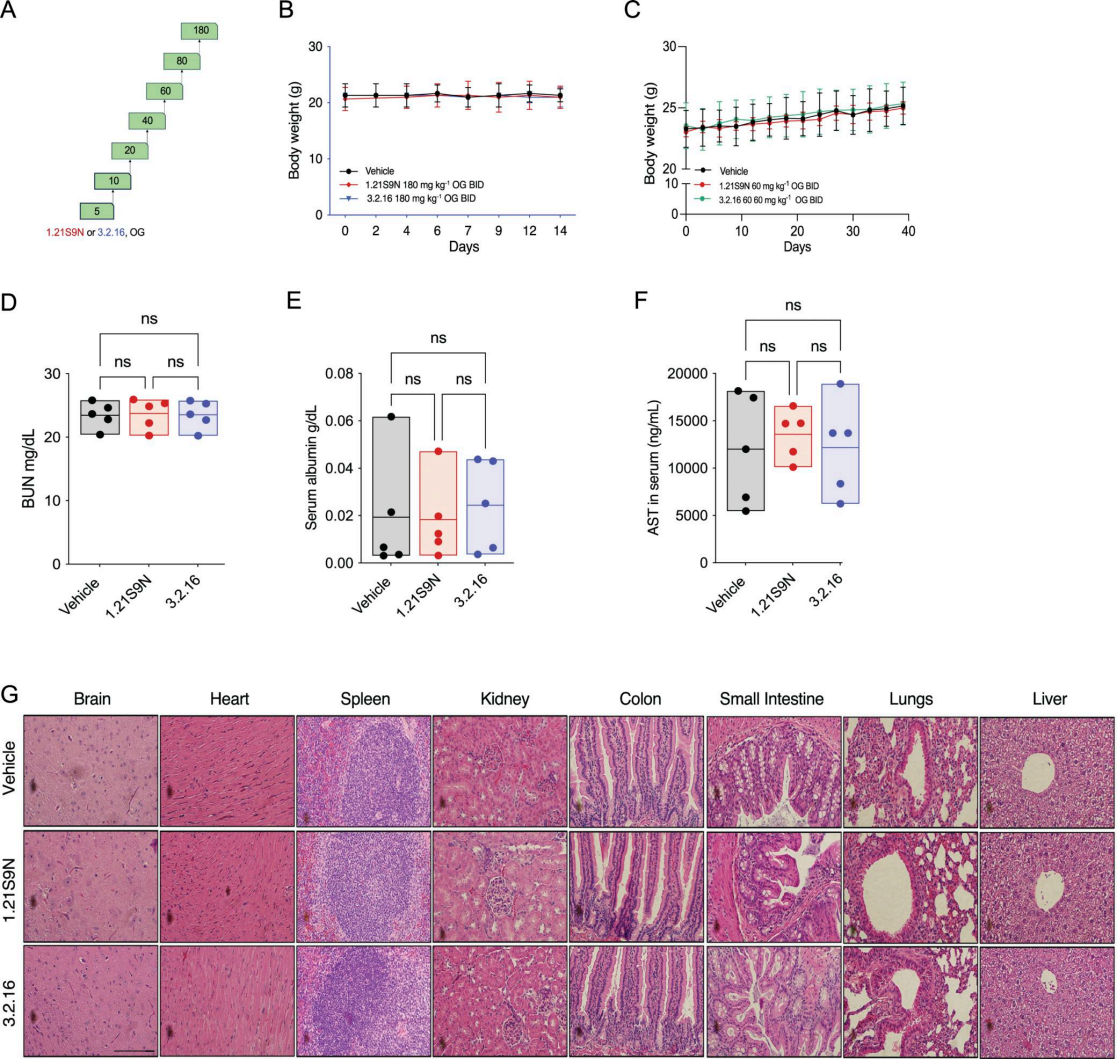
**Toxicology studies of dual** **HIFi** **. (A)** Mice were administered vehicle or HIFi (1.21S9N or 3.2.16) at the indicated dose (in mg/kg OG) every 12 h for two doses followed by daily dose escalation. **(B)** Mice were administered vehicle or HIFi (180 mg/kg OG BID) for 14 days. BW was measured every 2–3 days (mean + SD, *n* = 3 mice per group). **(C)** Mice were treated with vehicle or either 1.21S9N or 3.2.16 (60 mg/kg OG BID) for 40 days. BW (mean ± SD; *n* = 5 mice) was measured every 2–3 days. **(D–F)** BUN (D), serum albumin (E), and serum AST (F) levels were determined on day 40. Data are presented as individual values and mean (*n* = 5); ns, no significant difference, one-way ANOVA with the Sidak post-test. **(G)** Mice were euthanized on day 40, and hematoxylin-and-eosin–stained sections of major organs were prepared. Representative images are shown. Scale bar, 100 μm.

Although the maximum tolerated dose (MTD) of 1.21S9N and 3.2.16 when administered IP was 40 mg/kg (Fig. S2, D and E), MTD was not reached at doses up to 180 mg/kg BID OG for 14 days (Fig. 10, A and B). Mice maintained stable BW and appearance, including grooming, posture, feeding, social interaction, and respiration. Administration of 60 mg/kg BID OG for 40 days also had no effect on appearance, behavior, or BW (Fig. 10 C). There were no significant between-group differences in blood urea nitrogen (BUN), serum albumin, or aspartate aminotransferase (AST) on day 40 (Fig. 10, D–F), indicating no treatment-related renal or hepatic injury. Hematoxylin-and-eosin–stained sections from brain, heart, spleen, kidney, colon, small intestine, lung, and liver of mice treated with HIFi showed no histological abnormalities (Fig. 10 G). Parenchymal architecture and cellular morphology were comparable to controls, with no evidence of vascular congestion, edema, fibrosis, atypia, inflammation, or necrosis; intestinal mucosal integrity and hepatic lobular organization were preserved. Together, these findings indicate HIFi therapy is well tolerated with no evidence of toxicity when administered for 14 days at six times the therapeutic dose or for 40 days at two times the therapeutic dose.

## Discussion

There are over 100 publications in which immunohistochemical analysis of tumor biopsies has established an association between HIF-1α or HIF-2α expression and patient mortality (Wicks and Semenza, 2022), including lung, colorectal, pancreatic, breast, and prostate cancer, which are the major causes of cancer mortality in the United States, accounting for >300,000 deaths annually (Siegel et al., 2024). The association between intratumoral HIF-1/2α expression and cancer mortality is indicative of a large body of data demonstrating key roles of HIFs in cancer progression (Cowman and Koh, 2022; Rankin et al., 2016; Sitkovsky et al., 2014; Wicks and Semenza, 2022; Yuan et al., 2024). The limited therapeutic efficacy of ICB reflects multiple mechanisms by which cancer cells evade detection and killing by immune cells, and many of these are mediated by HIF-1/2 target gene products (Chouaib et al., 2012; Semenza, 2021; Sitkovsky et al., 2014).

Our integrated biophysical and molecular data demonstrate that 1.21S9N and 3.2.16 bind directly to HIF-1α and HIF-2α. MST, by virtue of analyzing the direct interaction of HIFi with purified HIF-1α protein, provided the most sensitive measure of binding affinity, with K_d_ estimates of 2.2 and 47 nM for 1.21S9N and 3.2.16, respectively. Compared with surface plasmon resonance, which requires immobilization and can distort apparent affinity depending on surface chemistry, the His-NTA MST format maintains proteins in solution and better reflects native binding properties (Seidel et al., 2013). Co-IP assays revealed submicromolar IC_50_ values for direct inhibition of HIF-1/2 subunit heterodimerization. Immunoblot assays demonstrated that HIFi-induced HIF-1α degradation was dependent on the presence of the targeted protein domain, as well as ubiquitination and proteasome activity, but was not dependent on the prolyl hydroxylation that triggers O_2_-dependent degradation. It is noteworthy that PT2385 and belzutifan bind to HIF-2α and block dimerization with HIF-1β, but do not cause HIF-2α degradation, which implies that lack of dimerization is not sufficient to induce degradation. This is consistent with the finding that deletion of the bHLH or PAS-B domain, which prevents dimerization, did not cause degradation. It is possible that the binding of 1.21S9N or 3.2.16 to the bHLH or PAS-B subdomain of HIF-1/2α, respectively, induces an allosteric change that decreases interaction with HIF-1β and increases interaction with a ubiquitin ligase. Experimental validation of this hypothesis will be challenging as there are over 600 known E3 ligases (Zheng and Shabek, 2017).

We demonstrated submicromolar inhibition of HIF-1/2 target gene expression in dozens of cancer cell lines. Administration of 1.21S9N or 3.2.16 to mice induced profound changes in the intratumoral expression of key regulators of innate and adaptive immunity, and reprogrammed the TIME from one dominated by immunosuppressive MDSCs and TAMs to one dominated by NK and T cells capable of mediating antitumor immunity. The decreased expression of mRNAs encoding the immune checkpoint proteins B7h3, Lag-3, Pd-l1, and Tim-3 in response to HIFi therapy was striking and represents the functional equivalent of administering multiple ICB antibodies. Thus, HIFi therapy both increased T/NK cell numbers and decreased T/NK cell exhaustion. The increased CR to ICB associated with HIFi therapy across a broad sampling of cancer types suggests that this combination will have widespread clinical utility. In mice that responded to combination therapy, the immune system continued to monitor for cancer recurrence after termination of therapy and responded to rechallenge such that the animals remained tumor-free.

Treatment of CRC tumor-bearing mice with HIFi had a profound antiangiogenic effect, with a markedly decreased luminal diameter of tumor blood vessels that resulted from the decreased expression of multiple mRNAs encoding angiogenic factors. Treatment of HNSCC tumor-bearing mice with HIFi led to major changes in the invasive properties of the tumors such that they were easily and cleanly excised, whereas tumors from vehicle-treated mice showed extensive infiltration of the surrounding tissue making clean excision difficult. Thus, dual HIF-1/2 inhibition has major effects on multiple critical aspects of cancer progression. The powerful effects of HIFi on angiogenesis, immune evasion, and tissue invasion reflect the inhibition of hundreds of HIF-1/2 target genes in both cancer and stromal cells within the tumor.

Administration of 1.21S9N or 3.2.16 at 60 mg/kg BID OG for 40 days was well tolerated with no change in animal appearance, behavior, or BW. In addition, histological analysis across eight major organs revealed no detectable treatment-related lesions. The absence of inflammatory, degenerative, or necrotic changes, together with stable BW and unchanged BUN, serum albumin, and AST values, suggests that there is a therapeutic window for effective HIF-1/2 inhibition in severely hypoxic cancer and stromal cells without adverse effects in other body tissues with normal O_2_ levels.

The carotid body (CB) senses hypoxemia and stimulates ventilation, a fundamental homeostatic response to hypoxia, which is inhibited by PT2385 (Cheng et al., 2020). HIF-2α is essential for CB development and function (Macias et al., 2018). Mice that are heterozygous for a HIF-2α null allele have significantly increased apnea episodes (Peng et al., 2011). In mice exposed to intermittent hypoxia, HIF-2α levels in the CB decreased (Nanduri et al., 2009), whereas HIF-1α levels increased (Peng et al., 2006), suggesting that HIF-1α and HIF-2α may have opposing effects in the CB. In clinical trials, an adverse effect of PT2977/belzutifan has been hypoxemia (Fallah et al., 2024), suggesting that selective HIF-2α inhibition may have adverse effects on ventilation that are not observed when both HIF-1α and HIF-2α are targeted.

The HIFi compounds we identified were surprising in several regards. First, SS1.21 and 1.21S9N bind to the bHLH domain of HIF-1α and HIF-2α, which share 82% identity and 90% similarity (HIF-1α amino acid residues 10–71). We are not aware of any other small molecule that targets the bHLH domain in any transcription factor. Second, SS3.2 and 3.2.16 bind to the PAS-B subdomain of HIF-1α and HIF-2α, which share 77% identity and 91% similarity (HIF-1α residues 235–298). While other compounds (PT2385 and PT2977) bind to the PAS-B subdomain of HIF-2α, those compounds do not bind to HIF-1α and do not induce HIF-2α degradation (Wallace et al., 2016). Thus, the interaction of SS3.2 or 3.2.16 with the PAS-B subdomain differs from that of PT2385 or PT2977. The existence of multiple HIF inhibitors with distinct molecular binding sites or binding modes will be advantageous in the therapeutic response to acquired drug resistance that might arise from mutations affecting the drug binding site, as exemplified by the multiple generations of BCR-ABL inhibitors that are used to treat leukemia (Alves et al., 2021).

## Materials and methods

### Study design and approvals

Sample size was *n* = 3 for analysis of RNA expression in cultured cells and *n* = 5 mice for tumor studies, except for flow cytometry studies where *n* = 5–10, with lesser number due to technical failure with one antibody (anti-CD3); these were the only data excluded in the study. All outliers were included. Endpoint: tumor-bearing mice were euthanized after 5 days of treatment or when tumor diameter exceeded 1.5 cm; the tumor became ulcerated; or the animal showed any sign of distress. The objective of the study was to identify small-molecule HIF inhibitors and demonstrate their effect on tumor growth alone and in combination with ICB. All mice were randomized prior to treatment, and tumor dimensions were measured in a blinded manner. Procedures involving mice were conducted in accordance with the National Institutes of Health (NIH) *Guide for the Care and Use of Laboratory Animals* (National Research Council, 2011). Protocols were reviewed and approved by the Johns Hopkins University Animal Care and Use Committee.

### SILCS screen

SILCS simulations (Guvench and MacKerell, 2009; Raman et al., 2011) were performed using the crystal structure of the HIF-2α:HIF-1β complex (PDB 4ZP4; Wu et al., 2015). The HIF-2α protein structure, modeled to generate coordinates of missing residues in the crystal structure (residues 11–25, 150–161, 202–218) using Modeller (Fiser and Sali, 2003), was immersed in an aqueous solution of solutes at ∼0.25 M representative of different functional groups (benzene, propane, formamide, methanol, dimethyl ether, imidazole, acetate, and methylammonium representative of aromatic, aliphatic, hydrogen bond donor, hydrogen bond acceptors, and negatively and positively charged groups). The solutes and water were subjected to oscillating excess chemical potential (μ_ex_) Grand Canonical Monte Carlo/Molecular Dynamics (GCMC/MD) simulations (Lakkaraju et al., 2014) to sample the distribution of solutes and water in and around the protein.

From these simulations, probability distributions of solutes and water were determined by counting their occurrence on grid points defined as 1 Å^3^ voxels that encompass the protein structure, normalized to the concentration of solutes in aqueous solution, and Boltzmann-transformed (Lakkaraju et al., 2015) to yield 3D distributions of GFEs of functional groups around the protein, termed GFE FragMaps. GCMC/MD simulations were performed using in-house GCMC code (Lakkaraju et al., 2015) with MD performed using the Gromacs package (Abraham et al., 2015). Force field parameters were the CHARMM36m protein force field (Huang et al., 2017) with the CHARMM TIP3P water model (Jorgensen, 1981) and the CHARMM General Force Field (CGenFF; Vanommeslaeghe et al., 2010) used for the solutes, fragments, and ligands (Goel et al., 2021; Ustach et al., 2019).

Identification of putative ligand binding sites on HIF-2α utilized the SILCS-Hotspots approach (MacKerell et al., 2020). A set of small mono- and bicyclic ring fragments that commonly occur in known drugs (Taylor et al., 2014) were docked using the SILCS-MC approach into the FragMaps encompassing the entire protein structure. Spatial clustering was performed for individual fragments followed by clustering over all fragment types to identify binding sites encompassing one or more fragments, called Hotspots. Three putative binding sites were chosen based on the presence of two or more adjacent Hotspots, along with FragMaps, protein surface, and exclusion map data.

Pharmacophore features were generated at each site using the SILCS-Pharm protocol (Yu et al., 2015). Virtual database screening was performed by selecting multiple pharmacophore hypotheses for each site that contained a subset of three or four of the total number of features in each site in various spatial 3D relationships. The three- and four-point pharmacophore hypotheses were all individually screened against an in-house database that contains 54,359 compounds from Maybridge, 710,933 compounds from ChemBridge, and 3,114 FDA-approved drugs. For each individual pharmacophore hypothesis, all compounds with the correct number and type of functional groups that were spatially within a root-mean-square difference <1.2 Å of the features were considered hits with a maximum of 10,000 being selected. All hits from the individual pharmacophores for each site were then pooled for further analysis.

The hit compounds were subjected to free energy scoring based on the SILCS-MC approach to generate LGFE scores. Calculating LGFE involves classification of the ligand atoms into FragMap type, followed by the summation of GFEs at the coordinate position of the classified ligand atoms (Goel et al., 2021; Ustach et al., 2019). SILCS-MC involves sampling the orientation and conformation of the ligand in the field of the FragMaps, based on the LGFE score, along with the intramolecular energy of the ligand, based on the CGenFF function with a 1/4r dielectric constant (Vanommeslaeghe et al., 2010). SILCS-MC pose refinement was performed starting from the docked orientation of each ligand from the pharmacophore search. MC sampling involved 100 steps of translations, molecular rotations, and dihedral rotations of 0.5 Å, 15°, and 180°, respectively, followed by MC simulated annealing of 1,000 steps of 0.25 Å, 9°, and 9°, respectively, for a minimum of 50 runs based on a convergence of 0.5 kcal/mol up to a maximum of 250 runs. Five repeats of this protocol were performed at each site, and binding pose and orientation of ligands were ranked based on most negative LGFE scores.

Final compound selection considered physical properties indicative of drug-likeness. LogP(o/w), number of H-bond donors and acceptors, and molecular mass were obtained for the selected compounds using MOE (Chemical Computing Group). 4-dimensional bioavailability (4DBA) values (Oashi et al., 2011), which combine Lipinski’s rule of five (Lipinski, 2000) into a single scalar value, were calculated. Ligands with molecular weight <200 and >550 Da were discarded. Ligands that did not pass the PAINS rules (Baell and Holloway, 2010) indicating potential toxicity were also removed. The remaining ligands were grouped into clusters based on chemical similarity using Morgan fingerprinting (cutoff = 0.45) with compounds for biological evaluation obtained by choosing ligands with the most negative LGFE scores in each cluster. Virtual database screening was performed targeting each site involving first the pharmacophore screening followed by final ranking based on the SILCS-MC LGFE scores. For each site, the top 1,000 ranked ligands from each database were selected for final filtering based on physical properties, including Lipinski’s rule of five and the 4DBA metric. From this procedure, the top 100 compounds were selected for each site.

### Cell culture

The source of cell lines and culture media are shown in Table S1. Cell line authentication and *Mycoplasma* testing were performed at the Johns Hopkins University Genetic Resources Core Facility. Other cell culture reagents are shown in Table S2.

### MST

Purified recombinant His_6_-tagged HIF-1α (Table S2) was thawed on ice and exchanged into MST buffer A (adapted from Woodfield et al. [2022]): 150 mM NaCl, 10 mM MgCl_2_, 2 mM dithiothreitol (DTT), and 0.1% Tween-20. Monolith His-tag Labelling Kit RED2-Tris-NTA (NanoTemper Technologies) was incubated with His_6_-HIF-1α at room temperature on a shaker for 30 min followed by centrifugation for 1 min at 13,000 RPM to remove any insoluble material. As specified by the manufacturer, MST-optimized RED2-Tris-NTA dye with nanomolar affinity does not require purification after labeling. HIFi compounds were serially diluted (1:1) in MST buffer B (150 mM NaCl, 10 mM MgCl_2_, 0.5 mg/ml BSA, 40% glycerol, 2 mM DTT, and 0.1% Tween-20) 10–12 times. Dye–protein complex was added to each HIFi dilution for a final protein concentration of 1.4 μM. Monolith NT.115 capillaries (NanoTemper Technologies) were preflushed with MST buffer containing 0.05% Tween-20 and 0.05% BSA to reduce sticking. Measurements were acquired on a Monolith NT.115 instrument (NanoTemper Technologies) with red channel excitation (Cy5) using 20% LED power and 20% MST power. Binding curves, relative fluorescence, and fraction binding were fit to a 1:1 binding model (K_d_) using MO.Affinity Analysis version 2.3 software (NanoTemper Technologies).

### RT-qPCR assays

Cells were cultured in 6-well plates overnight (see Table S1 for seeding density), pretreated for 1 h with HIFi, exposed to 20% or 1% O_2_ for 24 h, and lysed in TRIzol (Table S2). The tumor tissue was flash-frozen in liquid nitrogen, and RNA was extracted using TRIzol. RNA was reverse-transcribed to cDNA using High Capacity RNA-to-cDNA Kit, and qPCR was performed using SYBR Green qPCR Master Mix and the CFX96 Real-Time PCR Detection System (Table S2), using primers listed in Table S3. The expression of target mRNA (E) was normalized to 18S rRNA using the threshold cycle (Ct) method and expressed as fold change: E = 2^–Δ(ΔCt)^, where ΔCt = Ct_target gene_ – Ct_18S rRNA_ and Δ(ΔCt) = ΔCt_treatment_ – ΔCt_control_. To determine IC_50_ values, we analyzed the expression of several HIF target genes in each cell line and chose the gene with highest fold induction when that cell line was exposed to 1% O_2_ for 24 h in the absence of HIFi.

### Co-IP assays

Soluble lysates were prepared from cultured cells in lysis buffer (50 mM HEPES [pH 7.9], 150 mM NaCl, 1 mM EDTA, 10% glycerol, 1% IGEPAL, 1 mM PMSF supplemented with cOmplete protease inhibitor cocktail), and debris was pelleted by centrifugation at 13,000 RPM for 20 min. The supernatant was precleared by incubation with protein G Sepharose (Table S2) for 1 h, an aliquot was reserved for input analysis, and remaining lysate was incubated with antibody overnight at 4°C. Protein G Sepharose was added to the samples and incubated for 4 h at 4°C. Immunoprecipitates were washed three times with lysis buffer and subjected to immunoblot assay using antibodies listed in Table S2. To test whether HIFi disrupted dimerization, a two-lysate Co-IP assay was performed under nondenaturing conditions. Cells were transiently transfected with expression vector encoding HIF-1α, HIF-2α, or HIF-1β. Cells were lysed separately in 20 mM Tris-HCl (pH 7.5), 150 mM NaCl, 0.5% NP-40, 1 mM EDTA, supplemented with protease inhibitors. Lysates were clarified at 14,000×*g* for 10 min at 4°C. The HIF-1α or HIF-2α lysate was treated with HIFi or vehicle for 30 min at 4°C, then mixed with the HIF-1β lysate, and incubated for 1 h at 4°C. Co-IP was performed by the addition of 2 µg of anti-HIF-1β antibody prebound to protein A/G magnetic beads (Thermo Fisher Scientific) overnight at 4°C. Beads were washed three times with: (1) 20 mM HEPES (pH 7.4), 150 mM NaCl, 0.5% NP-40, 1 mM EDTA; and (2) 10 mM sodium phosphate (pH 7.3), 150 mM NaCl, 0.5% NP-40, 1 mM EDTA. Bound proteins were eluted in Laemmli buffer and separated by SDS–PAGE, and immunoblot assays were performed.

### Immunoblot assays

Cultured cells or tumor tissue was lysed in radioimmunoprecipitation assay buffer (Table S2). Proteins were fractionated by 8–10% SDS–PAGE and transferred to nitrocellulose membranes, which were blocked with 5% (wt/vol) nonfat milk/0.1% Tween-20 in PBS for 1 h and incubated at 4°C overnight with primary antibody (Table S2) in blocking solution. Membranes were incubated with HRP-conjugated secondary antibody for 1 h. The chemiluminescent signal was generated using ECL Prime (Table S2) and detected using ChemiDoc Imaging System (Bio-Rad).

### Tumor studies

We selected tumor × HIFi × ICB models based on the following criteria: (1) robust inhibition of HIF target gene expression in the cell line by the HIFi as documented in Fig. 2 P; and (2) published work indicating the tumor model was resistant to the chosen ICB. Cancer cells were trypsinized, rinsed with PBS, and injected into mice in 100 μl of a 1:1 Matrigel:PBS suspension (Table S2). The number of cells injected, site of injection, and strain/sex of recipient mice are shown in Table S4. Tumor long (L) and short (S) axes were measured (in mm) using electronic calipers, and tumor volume (V) was calculated: V (mm^3^) = (L × S^2^)/2. Drug stocks (400–800 mg/ml in DMSO) were formulated for IP or OG dosing at 10–180 mg/kg as described in Table S5. Mice were euthanized when tumor diameter reached 1.5 cm, tumors became ulcerated, or mice showed signs of distress. For rechallenge studies, cells were implanted in the adjacent MFP or contralateral flank relative to the initial injection.

### Measurement of breathing

Breathing was monitored by whole-body plethysmograph (SCIREQ, Montreal, Canada) in unsedated 2-month-old male C57BL/6 mice (Jackson Laboratory) breathing room air as described previously (Nanduri et al., 2009; Peng et al., 2011). All measurements were made between 1:30 p.m. and 3:30 p.m. to exclude any confounding influence of circadian variations. All measurements were made at an ambient temperature of 25 ± 1°C. Starting 4 h after the last drug dose, 1 h was allowed for acclimation of mice to the plethysmography chamber, and breathing was then monitored continuously for 2 h. Apnea was defined as cessation of breathing longer than the duration of three normal breaths. Apneas were scored with a commercial program (IOX, SCIREQ). Sniffs, postsigh apneas, and movement artifacts were excluded from analysis.

### Flow cytometry

Tumors were minced and digested using collagenase (1 mg/ml) at 37°C for 30 min. The resulting single-cell suspension was passed through a 70-μm strainer and rinsed twice with cold PBS. Cells were resuspended in Fc Block (BD Biosciences) and stained with multiple fluorescent antibodies (Table S6) to characterize specific immune cell populations (Salman et al., 2022; Samanta et al., 2020). Cells were processed for flow cytometry analysis using FACSDiva software (BD Biosciences). Live cells were identified and gated based on side-scatter and forward-scatter plots. Gating strategies were employed using unstained control, fluorescence minus one, and single-stained cell samples (Fig. S4 R). FlowJo software was used for data analysis.

### Tumor immunohistochemistry

Tumor tissues were fixed in 10% formalin and paraffin embedded for sectioning. Tissue sections were deparaffinized, hydrated, and immersed in antigen retrieval solution (Table S2). Sections were blocked with 10% normal goat serum for 1 h; incubated with anti-CD31 antibody overnight at 4°C; treated with 3% H_2_O_2_ for 10 min; incubated with SignalStain Boost Detection Reagent for 30 min at room temperature; washed; and covered with SignalStain DAB (Cell Signaling Technology) followed by hematoxylin counterstaining. Sections were dehydrated and mounted with coverslips. For analysis, three fields at 20× magnification from three different tumors were captured using ImageJ (NIH).

### Toxicity study

C57BL/6 mice (The Jackson Laboratory) were randomized into treatment groups. HIFi was administered at 60 mg/kg OG BID for 40 days. Animals were monitored for behavior, posture, gait, grooming, and signs of distress. On day 40 (4 h after last dose), mice were euthanized, and gross necropsy was performed. Major organs were collected, fixed in 10% neutral buffered formalin, processed routinely, paraffin-embedded, sectioned at 4–5 μm, and stained with hematoxylin and eosin, and representative photomicroscopic images were obtained. Whole blood was collected via terminal cardiac puncture, allowed to clot, and centrifuged to isolate serum. BUN (mg/dl), serum albumin (g/dl), and AST (U/l) were measured using ELISA kits (Table S2) according to the manufacturer’s instructions.

### Pharmacokinetic analysis

Male C57BL/6 mice were administered a single dose of HIFi, either IP (40 mg/kg) or OG (60 mg/kg). Blood samples were obtained at 1, 2, 4, 8, 12, 16, and 24 h after dose. Plasma was isolated by centrifugation (1,500×*g*, 10 min, 4°C) and stored at −80°C until analysis. HIFi concentration in plasma was quantified using a validated LC-MS/MS assay (LLOQ = 1 ng/ml). Calibration standards and quality controls were prepared in matched matrices.

### Statistical analysis

All analyses were performed using Prism 9 (GraphPad Software). Significant differences between groups were determined using Student’s *t* test or analysis of variance with multiple comparisons. Kaplan–Meier survival plots were analyzed by the log-rank test.

### Online supplemental material

Fig. S1 shows molecular analyses and is related to Figs. 1, 2, and 3. Fig. S2 shows HIFi concentration-dependent in vitro and dose-dependent in vivo effects. Fig. S3 shows CRC, HNSCC, and BrCa tumor models and is related to Figs. 4 and 5. Fig. S4 shows melanoma, prostate and breast tumor models and is related to Figs. 6 and 7. Fig. S5 shows analysis of mice treated with1.21S9N or 3.2.16 and is related to Fig. 8. Table S1 shows the cancer cell lines used in vitro. Table S2 shows antibodies used in this study. Table S3 shows oligonucleotides used in this study. Table S4 shows cancer cell lines and mouse strains used in vivo. Table S5 shows the formulation of drugs administered in vivo. Table S6 shows antibodies used for flow cytometry.

## Ethics declarations

A.D. MacKerell, A. Kumar, G.L. Semenza, S. Salman, and Y. Hwang are inventors on US provisional patent application no. 63/669,986. G.L. Semenza is cofounder of and holds equity in HIF Therapeutics, Inc., and this arrangement has been reviewed and approved by Johns Hopkins University in accordance with its conflict-of-interest policies. A.D. MacKerell is cofounder and CSO of SilcsBio LLC, and this arrangement has been reviewed and approved by the University of Maryland, Baltimore, in accordance with its conflict-of-interest policies.

## Supplementary Material

Table S1 shows cancer cell lines used in this study.

Table S2 shows antibodies against human or mouse proteins used for immunoblot assays, immunohistochemistry, immunoprecipitation, or tumor studies; and other reagents for cell culture, gene expression, and MST (ELISA).

Table S3 shows PCR primer pairs (forward [F] and reverse [R]) used for analysis of human and mouse mRNA levels in cell lines and tumor tissue.

Table S4 shows cancer cell lines and mouse strains used for tumor studies.

Table S5 shows formulation of drugs administered in vivo.

Table S6 shows antibodies used for flow cytometry.

SourceData F2 is the source file for Fig. 2.

SourceData F3 is the source file for Fig. 3.

SourceData F4 is the source file for Fig. 4.

SourceData F5 is the source file for Fig. 5.

SourceData FS3 is the source file for Fig. S3.

SourceData FS5 is the source file for Fig. S5.

## Data Availability

All of the data are available in the main text or the online supplemental material. The SILCS FragMaps and pharmacophore models used for the identification of dual HIFi, as well as the raw biophysical datasets, are available from the corresponding authors upon request. Source data for the immunoblots are provided in the source data files.
