# Supplementary material for: Targeting conserved domains of hypoxia-inducible factors for cancer therapy
Source: J Exp Med. 2026 Apr 2;223(5):e20251009. doi: 10.1084/jem.20251009 (PMC13068195; doi:10.1084/jem.20251009)
Supplement: Table S1 — shows cancer cell lines used in this study. [file jem_20251009_tables1.docx]

Table S1. Cancer cell lines used in this study.

| **Cell line**  (Seeding density/well  in 6-well plate) | **Catalog number** | **Source** | **Complete medium*^,#^** |
| --- | --- | --- | --- |
| MDA-MB-231 (3x10^5^) | HTB-26 | ATCC | DMEM + 10% FBS + 1% [P/S](https://www.thermofisher.com/order/catalog/product/15140122) |
| SUM149PT (3.5x10^5^) | HCL-SUM149 | BIOIVT | Ham's F-12 + 10mM HEPES + 1 μg/ml  hydrocortisone + 5% FBS + 1% P/S |
| SUM159 (2x10^5^) | HCL-SUM159 | BIOIVT | DMEM/F-12 +10% FBS + 1% P/S |
| MCF7 (2x10^5^) | HTB-22 | ATCC | DMEM + 10% FBS + 1% [P/S](https://www.thermofisher.com/order/catalog/product/15140122) |
| T-47D (2x10^5^) | HTB-133 | ATCC | RPMI-1640 + 10% FBS + 1% [P/S](https://www.thermofisher.com/order/catalog/product/15140122) |
| ZR-75-1 (2x10^5^) | ZR-75-1 | ATCC | RPMI-1640 + 10% FBS + 1% [P/S](https://www.thermofisher.com/order/catalog/product/15140122) |
| BT-474 (2.5x10^5^) | HTB-20 | ATCC | HybriCare + 10% FBS + 1% [P/S](https://www.thermofisher.com/order/catalog/product/15140122) |
| HCC1954 (2.5x10^5^) | CRL-4338 | ATCC | RPMI-1640 + 10% FBS + 1% [P/S](https://www.thermofisher.com/order/catalog/product/15140122) |
| HCT116 (2.5x10^5^) | CCL-247 | ATCC | McCoy's 5A + 10% FBS + 1% [P/S](https://www.thermofisher.com/order/catalog/product/15140122) |
| DLD-1 (2.5x10^5^) | CCL-221 | ATCC | RPMI-1640 + 10% FBS + 1% [P/S](https://www.thermofisher.com/order/catalog/product/15140122) |
| U-87 MG (3x10^5^) | HTB-14 | ATCC | EMEM + 10% FBS + 1% [P/S](https://www.thermofisher.com/order/catalog/product/15140122) |
| FaDu (5x10^5^) | HTB-43 | ATCC | EMEM + 10% FBS + 1% [P/S](https://www.thermofisher.com/order/catalog/product/15140122) |
| A549 (2.5x10^5^) | CCL-185 | ATCC | DMEM + 10% FBS + 1% [P/S](https://www.thermofisher.com/order/catalog/product/15140122) |
| A-375 (3x10^5^) | CRL-1619 | ATCC | DMEM + 10% FBS + 1% [P/S](https://www.thermofisher.com/order/catalog/product/15140122) |
| OVCAR-3 (3.5x10^5^) | HTB-161 | ATCC | RPMI-1640 + 1 ug/mL INS + 20% FBS  + 1% [P/S](https://www.thermofisher.com/order/catalog/product/15140122) |
| LNCaP (5x10^5^) | CRL-1740 | ATCC | RPMI-1640 + 10% FBS + 1% [P/S](https://www.thermofisher.com/order/catalog/product/15140122) |
| C4-2B (3.5x10^5^) | CRL-3314 | ATCC | RPMI-1640 + 10% FBS + 1% [P/S](https://www.thermofisher.com/order/catalog/product/15140122) |
| DU145 (3.5x10^5^) | HTB-81 | ATCC | RPMI-1640 + 10% FBS + 1% [P/S](https://www.thermofisher.com/order/catalog/product/15140122) |
| PC-3 (3.5x10^5^) | CRL-1435 | ATCC | RPMI-1640 + 10% FBS + 1% [P/S](https://www.thermofisher.com/order/catalog/product/15140122) |
| Hep3B (2.5x10^5^) | HB-8064 | ATCC | DMEM + 10% FBS + 1% P/S |
| 4T1 (2x10^5^) | CRL-2539 | ATCC | RPMI-1640 + 10% FBS + 1% [P/S](https://www.thermofisher.com/order/catalog/product/15140122) |
| EMT6 (2x10^5^) | CRL-2755 | ATCC | Waymouth's + 2 mM L-Gln + 15% FBS  + 1% P/S |
| E0771 (2x10^5^) | CRL-3461 | ATCC | DMEM + 20 mM HEPES + 10% FBS  + 1% P/S |
| CT26 (2.5x10^5^) | CRL-2638 | ATCC | RPMI-1640 + 10% FBS + 1% [P/S](https://www.thermofisher.com/order/catalog/product/15140122) |
| Hepa1-6 (2.5x10^5^) | CRL-1830 | ATCC | DMEM + 10% FBS + 1% P/S |
| LLC1 (2.5x10^5^) | CRL-1642 | ATCC | DMEM + 10% FBS + 1% P/S |
| B16-F10 (2x10^5^) | CRL-6475 | ATCC | DMEM + 10% FBS + 1% P/S |
| MyC-CaP (2.5x10^5^) | CRL-3255 | ATCC | DMEM + 10% FBS + 1% P/S |
| TRAMP-C2 (3.5x10^5^) | CRL-2731 | ATCC | DMEM + 5% Nu-Serum IV + 5 µg/mL  INS + 10 nM DHT + 5% FBS + 1% P/S |
| RM-1 (3.5x10^5^) | CRL-3310 | ATCC | DMEM + 10% FBS + 1% P/S |
| GL261 (2.5x10^5^) | Gift | D. Zagzag | DMEM + 10% FBS + 1% P/S |
| MDA-MB-231 BrM2 (3x10^5^) | Gift | J. Massagué | DMEM + 10% FBS + 1% P/S |
| SUM159-M1a (2.5x10^5^) | Gift | Y. Kang | DMEM/F-12 +10% FBS+1% P/S |
| PANC-1 (2.5x10^5^) | Gift | H. Shukla | DMEM + 10% FBS + 1% P/S |
| KPC (2.5x10^5^) | Gift | H. Shukla | DMEM + 10% FBS + 1% P/S |
| SCC-VII (2.5x10^5^) | Gift | J. Grandis | DMEM + 10% FBS + 1% P/S |
| DX1 (3.5x10^5^) | Gift | D. Zhao | DMEM + 10% FBS + 1% P/S |
| NT2.5 (3.5x10^5^) | Gift | E. Jaffee | RPMI-1640 +1% NEAA + 1% Pyruvate  + 1% L-Gln + 20% FBS + 1% P/S |
| MC38 (2.5x10^5^) | Gift | J. Schneck | RPMI-1640 + 10% FBS + 1% [P/S](https://www.thermofisher.com/order/catalog/product/15140122) |
| ID8 (3.5x10^5^) | Gift | W. Zou | RPMI-1640 + 10% FBS + 1% [P/S](https://www.thermofisher.com/order/catalog/product/15140122) |

*Abbreviations: DHT, dihydrotestosterone; DMEM: Dulbecco’s modified Eagle medium; EMEM, Eagle’s minimal essential medium; FBS, fetal bovine serum; Gln, glutamine; INS, insulin; NEAA, non-essential amino acids; P/S, penicillin-streptomycin.

^#^All percentages represent v/v.
