# Supplementary material for: Targeting conserved domains of hypoxia-inducible factors for cancer therapy
Source: J Exp Med. 2026 Apr 2;223(5):e20251009. doi: 10.1084/jem.20251009 (PMC13068195; doi:10.1084/jem.20251009)
Supplement: Table S2 — shows antibodies against human or mouse proteins used for immunoblot assays, immunohistochemistry, immunoprecipitation, or tumor studies; and other reagents for cell culture, gene expression, and MST (ELISA). [file jem_20251009_tables2.docx]

**Table S2.** **Antibodies against human (h) or mouse (m) proteins used for immunoblot assays (IB), immunohistochemistry (IHC), immunoprecipitation (IP) or tumor studies (TS); and other reagents for cell culture (CC), gene expression (GE), microscale thermophoresis (MST), (ELISA)**

| **Reagent/Material** | **Catalog number** | **Source** | **App*** |
| --- | --- | --- | --- |
| HIF-1α antibody | 610959 | BD | IB |
| HIF-1α antibody | 10006421 | Cayman | IB |
| HIF-1β antibody | NB100-110 | Novus Biologicals | IB |
| HIF-2α antibody | NB100-122 | Novus Biologicals | IB |
| Ubiquitin antibody | MAB8595 | Novus Biologicals | IB |
| β-Actin antibody | Sc-47778 | Santa Cruz | IB |
| CD31/PECAM-1 antibody | NB100-2284 | Novus Biologicals | IHC |
| Antigen retrieval solution | 4955-58 | ThermoFisher | IHC |
| SignalStain Boost Detection Reagent | 8114 | Cell Signaling Tec | IHC |
| Syrian Hamster anti-mouse CTLA-4 | BP0131 | Bio X Cell | TS |
| Syrian Hamster IgG | BP0087 | Bio X Cell | TS |
| Rat IgG2a, anti-mouse PD-1 | BP0146 | Bio X Cell | TS |
| Rat IgG2a isotype control, anti-TNP | BE0089 | Bio X Cell | TS |
| Oxaliplatin | NDC 16729-332-05 | Accord Healthcare | TS |
| 0.9% Sodium Chloride, Saline | NDC 0409-488-10 | Hospira | TS |
| Matrigel Matrix Basement Membrane | 354234 | Corning | TS |
| TRIzol Reagent | 15596026 | Invitrogen | GE |
| MG132 | S2619 | Selleckchem | IB, IP |
| TAK-243 | MLN7243 | Selleckchem | IB |
| PT2385 | CS-4326 | Chemscene | GE |
| PT2977 | HY-125840 | MedChem Express | GE |
| PEG-400 | PX1286B-2 | Sigma | TS |
| Ethanol | 1009861000 | Sigma | TS |
| ECL Prime | RPN2232 | Cytiva | IB |
| Collagenase type I | SCR103 | Sigma Aldrich | TS |
| High-Capacity RNA-to-cDNA Kit | 4387406 | Applied Biosystem | GE |
| iQ SYBR Green Supermix | 1708884 | BioRad | GE |
| Dual Luciferase Reporter Assay System | E1960 | Promega | CC |
| HEPES | 15-630-080 | ThermoFisher | IB |
| RIPA buffer | 8900 | ThermoFisher | IB |
| Protease inhibitor cocktail | P8340 | Sigma | IB |
| Triton X-100 | 11332481001 | Sigma | IB |
| EDTA | 60-00-4 | Sigma | IB |
| Tris-HCl | 10812846001 | Sigma | IB |
| Glycerol | G5516 | Sigma | IB |
| protein G-Sepharose | 17061801 | Sigma | IP |
| SDS | 151-21-3 | Sigma | IB |
| Sodium pyruvate | 11360070 | ThermoFisher | CC |
| L-glutamine | 25030081 | ThermoFisher | CC |
| Dulbecco’s modified Eagle’s medium (DMEM) | 10-013-CV | Corning | CC |
| Eagle's Minimum Essential Medium (EMEM) | 112-018-101 | Quality Biological | CC |
| Ham's F-12 | 10-080-CV | Corning | CC |
| DMEM/F-12 | 10-092-CV | Corning | CC |
| RPMI-1640 | 10-040-CV | Corning | CC |
| HybriCare | ATCC 46-X | ATCC | CC |
| McCoy's 5A | 10-050-CV | Corning | CC |
| Waymouth's | 11220-035 | Invitrogen | CC |
| HEPES | SH30237.01 | Cytiva | CC |
| Insulin | I9278-5ml | Sigma | CC |
| Nu-Serum IV | 355104 | Corning | CC |
| Dihydrotestosterone | S4757 | Selleck Chemicals | CC |
| Fetal Bovine Serum (FBS) | A3160602 | ThermoFisher | CC |
| Penicillin-Streptomycin | P0781 | Sigma | CC |
| Recombinant His_6_-tagged HIF-1α | ab154478 | Abcam | MST |
| RED2-Tris-NTA dye | MO-L018 | Nanotemper | MST |
| Urea Assay Kit | NBP3-24543 | Novus Biologicals | ELISA |
| Mouse aspartate aminotransferase | NBP2-69877 | Novus Biologicals | ELISA |
| Mouse serum albumin | NBP3-38952 | Novus Biologicals | ELISA |

*Abbreviations: APP, application; CC, cell culture; ELISA, enzyme-linked immunosorbent assay; GE, gene expression; IB, immunoblot; IP, immunoprecipitation; MST, microscale thermophoresis; TS, tumor study.
