# Supplementary material for: Targeting conserved domains of hypoxia-inducible factors for cancer therapy
Source: J Exp Med. 2026 Apr 2;223(5):e20251009. doi: 10.1084/jem.20251009 (PMC13068195; doi:10.1084/jem.20251009)
Supplement: Table S3 — shows PCR primer pairs (forward [F] and reverse [R]) used for analysis of human and mouse mRNA levels in cell lines and tumor tissue. [file jem_20251009_tables3.docx]

**Table S3. PCR primer pairs (forward [F] and reverse [R]) used for analysis of human (H) and mouse (m) mRNA levels in cell lines and tumor tissue.**

| **Oligonucleotide** | **Sequence (5’ to 3’)** | **Source** | **Application** |
| --- | --- | --- | --- |
| H-ANGPTL4-F | GGACACGGCCTATAGCCTG | Sigma | RT-qPCR |
| H-ANGPTL4-R | CTCTTGGCGCAGTTCTTGTC | Sigma | RT-qPCR |
| H-PGF-F | GAACGGCTCGTCAGAGGTG | Sigma | RT-qPCR |
| H-PGF-R | ACAGTGCAGATTCTCATCGCC | Sigma | RT-qPCR |
| H-CA9-F | TCTCGTTTCCAATGCACGTACAGC | Sigma | RT-qPCR |
| H-CA9-R | AGTGACAGCAGCAGTTGCACAGT | Sigma | RT-qPCR |
| H-EPO-F | GGAGGCCGAGAATATCACGAC | Sigma | RT-qPCR |
| H-EPO-R | CCCTGCCAGACTT CTACGG | Sigma | RT-qPCR |
| H-NDRG1-F | CTCCTGCAAGAGTTTGATGTCC | Sigma | RT-qPCR |
| H-NDRG1-R | CATGCCGATGTCATGGTAGG | Sigma | RT-qPCR |
| H-PPFIA4-F | ATGCTGCCACTGGTTACACG | Sigma | RT-qPCR |
| H-PPFIA4-R | CTCTGCGGATGTTGTCTCCC | Sigma | RT-qPCR |
| H-ADM-F | ATGAAGCTGGTTTCCGTCG | Sigma | RT-qPCR |
| H-ADM-R | GACCATCCGCAGTTCCCTCTT | Sigma | RT-qPCR |
| H-RPL13A-F | CTCAAGGTCGTGCGTCTG | Sigma | RT-qPCR |
| H-RPL13A-R | TGGCTTTCTCTTTCCTCTTCTC | Sigma | RT-qPCR |
| H-18S-rRNA-F | GTAACCCGTTGAACCCCATT | Sigma | RT-qPCR |
| H-18S-rRNA-R | CCATCTCGGCTTATCCAACAATGA | Sigma | RT-qPCR |
| m-Ca9-F | TGCTCCAAGTGTCTGCTCAG | Sigma | RT-qPCR |
| m-Ca9-R | CAGGTGCATCCTCTTCACTGG | Sigma | RT-qPCR |
| m-Pgf-F | TCTGCTGGGAACAACTCAACA | Sigma | RT-qPCR |
| m-Pgf-R | GTGAGACACCTCATCAGGGTAT | Sigma | RT-qPCR |
| m-Adm-F | GGCGCTAAGTCGTGGGAAGA | Sigma | RT-qPCR |
| m-Adm-R | CTTCGCTCTGATTGCTGGCTTG | Sigma | RT-qPCR |
| m-Ndrg1-F | TCACGACATCGGCATGAACC | Sigma | RT-qPCR |
| m-Ndrg1-R | TTGTTGAGTGCGAACGGGTC | Sigma | RT-qPCR |
| m-Rpl13a-F | GGGCAGGTTCTGGTATTGGAT | Sigma | RT-qPCR |
| m-Rpl13a-R | GGCTCGGAAATGGTAGGGG | Sigma | RT-qPCR |
| m-18S-F | GTAACCCGTTGAACCCCATT | Sigma | RT-qPCR |
| m-18S-R | CCATCCAATCGGTAGTAGCG | Sigma | RT-qPCR |
| m-B7h3-F | ATGCTTCGAGGATGGGGTG | Sigma | RT-qPCR |
| m-B7h3-R | CCAGGCTCTGGGGAAAAGG | Sigma | RT-qPCR |
| m-Glut1-F | ATTGGCTCCGGTATCGTCAAC | Sigma | RT-qPCR |
| m-Glut1-R | GCTCAGATAGGACATCCAGGGTA | Sigma | RT-qPCR |
| m-Cd47-F | TGGTGGGAAACTACACTTGCG | Sigma | RT-qPCR |
| m-Cd47-R | CGTGCGGTTTTTCAGCTCTAT | Sigma | RT-qPCR |
| m-Cd73-F | CCTGCACACAAACGACGTG | Sigma | RT-qPCR |
| m-Cd73-R | CTGGTCTCCGGCATCCAAAA | Sigma | RT-qPCR |
| m-Il6-F | TAGTCCTTCCTACCCCAATTTCC | Sigma | RT-qPCR |
| m-Il6-R | TTGGTCCTTAGCCACTCCTTC | Sigma | RT-qPCR |
| m-Pdl1-F | GACCAGCTTTTGAAGGGAATG | Sigma | RT-qPCR |
| m-Pdl1-R | CTGGTTGATTTTGCGGTATGG | Sigma | RT-qPCR |
| m-Lag-3-F | CTGGGACTGCTTTGGGAAG | Sigma | RT-qPCR |
| m-Lag-3-R | GGTTGATGTTGCCAGATAACCC | Sigma | RT-qPCR |
| m-Tim3-F | TCAGGTCTTACCCTCAACTGTG | Sigma | RT-qPCR |
| m-Tim3-R | GGGCAGATAGGCATTTTTACCA | Sigma | RT-qPCR |
| m-Vegfa-F | CTGCCGTCCGATTGAGACC | Sigma | RT-qPCR |
| m-Vegfa-R | CCCCTCCTTGTACCACTGTC | Sigma | RT-qPCR |
| m-Angptl-4-F | CATCCTGGGACGAGATGAACT | Sigma | RT-qPCR |
| m-Angptl-4-R | TGACAAGCGTTACCACAGGC | Sigma | RT-qPCR |
| m-Pd-1-F | ACCCTGGTCATTCACTTGGG | Sigma | RT-qPCR |
| m-Pd-1-R | CATTTGCTCCCTCTGACACTG | Sigma | RT-qPCR |
