# Supplementary material for: Targeting conserved domains of hypoxia-inducible factors for cancer therapy
Source: J Exp Med. 2026 Apr 2;223(5):e20251009. doi: 10.1084/jem.20251009 (PMC13068195; doi:10.1084/jem.20251009)
Supplement: Table S4 — shows cancer cell lines and mouse strains used for tumor studies. [file jem_20251009_tables4.docx]

**Table S4. Cancer cell lines and mouse strains used for tumor studies.**

| **Cell line** | **Injected** | **Site** | **Mouse strain, sex** |
| --- | --- | --- | --- |
| HCT116 | 1 x 10^7^ | Flank | Athymic NCr-nu/nu, M |
| FaDu | 1 x 10^7^ | Flank | Athymic NCr-nu/nu, M |
| BT-474 | 1 x 10^7^ | MFP | BALB/c Nude, F |
| E0771 | 1 x 10^5^ | MFP | C57BL/6NCr, F |
| EMT6 | 1 x 10^5^ | MFP | BALB/cAnNCr, F |
| MC38 | 5 x 10^5^ | Flank | C57BL/6NCr, F |
| B16F10 | 5 x 10^5^ | Flank | C57BL/6NCr, F |
| KPC | 5 x 10^5^ | Flank | C57BL/6NCr, F |
| LLC | 5 x 10^5^ | Flank | C57BL/6NCr, F |
| DX1 | 1 x 10^6^ | Flank | C57BL/6NCr, M |
| SCCVII | 1 x 10^5^ | Flank | C3H/HeOuJ, M |
| CT26 | 3 x 10^5^ | Flank | BALB/cAnNCr, F |
| **Mouse strain** | **Catalog #** | **Source** | **Age/Sex** |
| NCI Athymic NCr-nu/nu | 553 | Charles River | 6-8 weeks old, Male |
| NCI BALB/cAnNCr | 555 | Charles River | 6-8 weeks old, Female |
| NCI C57BL/6NCr | 556 | Charles River | 6-8 weeks old, Female |
| NCI C57BL/6NCr | 556 | Charles River | 6-8 weeks old, Male |
| C57BL/6NCr | 664 | Jackson Laboratory | 2 month old, Male |
| BALB/c Nude | 194 | Charles River | 6-8 weeks old, Female |
| C3H/HeOuJ | 635 | Jackson Laboratory | 6-8 weeks old, Male |
| MMTV-PyMT | 2374 | Jackson Laboratory | 53 day old, Female |
