# Supplementary material for: Targeting conserved domains of hypoxia-inducible factors for cancer therapy
Source: J Exp Med. 2026 Apr 2;223(5):e20251009. doi: 10.1084/jem.20251009 (PMC13068195; doi:10.1084/jem.20251009)
Supplement: Table S5 — shows formulation of drugs administered in vivo. [file jem_20251009_tables5.docx]

**Table S5. Formulation of drugs administered in vivo.**

| **Formulation for IP administration** | | | | |
| --- | --- | --- | --- | --- |
| **Drug** | **Ethanol** | **PEG-400** | **Saline** | **Dose** |
| SS1.21 | 10% | 30% | 60% | 20 mg/kg |
| SS3.2 | - | 20% | 80% | 10 mg/kg |
| 1.21.S9N | 10% | 30% | 60% | 40 mg/kg |
| 3.2.16 | - | 40% | 60% | 40 mg/kg |
| Oxaliplatin | - | - | 100% | 10 mg/kg |
| α-CTLA-4 | - | - | 100% | 200 μg |
| α-PD-1 | - | - | 100% | 200 μg |
| **Formulation for OG administration** | | | | |
| **Drug** | **Ethanol** | **PEG-400** | **Water** | **Dose** |
| PT2385 | 5% | 45% | 50% | 30-60 mg/kg |
| 1.21S9N | 5% | 45% | 50% | 60-180 mg/kg |
| 3.2.16 | 5% | 45% | 50% | 60-180 mg/kg |
