# Supplementary material for: Targeting conserved domains of hypoxia-inducible factors for cancer therapy
Source: J Exp Med. 2026 Apr 2;223(5):e20251009. doi: 10.1084/jem.20251009 (PMC13068195; doi:10.1084/jem.20251009)
Supplement: Table S6 — shows antibodies used for flow cytometry. [file jem_20251009_tables6.docx]

**Table S6. Antibodies used for flow cytometry (FC).**

| **Antibodies** | **Catalog number** | **Source** | **Application*** |  |
| --- | --- | --- | --- | --- |
| CD45 | NB100-77417AF405 | Novus Biologicals | FC |  |
| CD45 | 103116 | BioLegend | FC |  |
| CD3 | NB100-64871PE | Novus Biologicals | FC |  |
| CD11b | NB110-89474PE | Novus Biologicals | FC |  |
| F4/80 | NB600-404APC | Novus Biologicals | FC |  |
| IFNγ | 505806 | BioLegend | FC |  |
| Ly6G | NBP2-53131APC | Novus Biologicals | FC |  |
| NK1.1 | NB100-77528-APC | Novus Biologicals | FC |  |
| PDL1 | 124312 | BioLegend | FC |  |
| **Cell Type*** | **Ab1*** | **Ab2*** | **Ab3*** | **Ab4*** |
| MDSC | Violet-CD45 | PE-CD11b | APC-Ly6G |  |
| TAM | Violet-CD45 | PE-CD11b | APC-F4/80 |  |
| NK cell | APC-Cy7-CD45 | PE-CD3 | APC-NK1.1 | FITC-IFNγ |
| Tumor cell | Violet-CD45 | PE-CD3 | APC-PDL1 |  |
| TIL | Violet-CD45 |  |  |  |
| T cell | Violet-CD45 | PE-CD3 |  |  |

*Abbreviations: Ab, antibody; APC, allophycocyanin; Cy7, cyanine 7; FITC, fluorescein isothiocyanate; MDSC, myeloid-derived suppressor cell; NK, natural killer; PE, phycoerythrin; TAM, tumor associated macrophage; TIL, tumor infiltrating leukocyte.
