# Supplementary figures and images for: Targeting conserved domains of hypoxia-inducible factors for cancer therapy
Source: J Exp Med. 2026 Apr 2;223(5):e20251009. doi: 10.1084/jem.20251009 (PMC13068195; doi:10.1084/jem.20251009)

Figure 2C

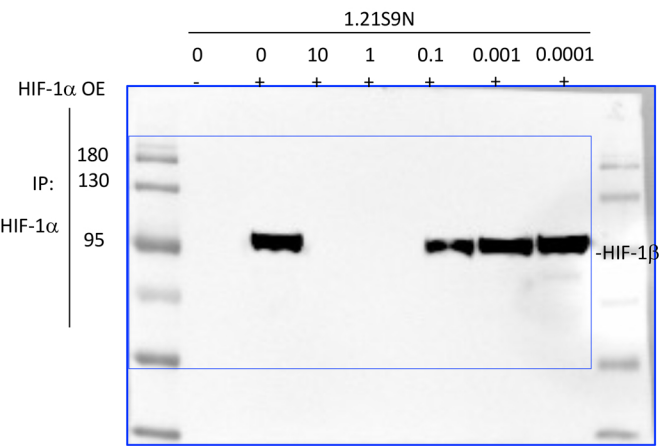

Figure 2E

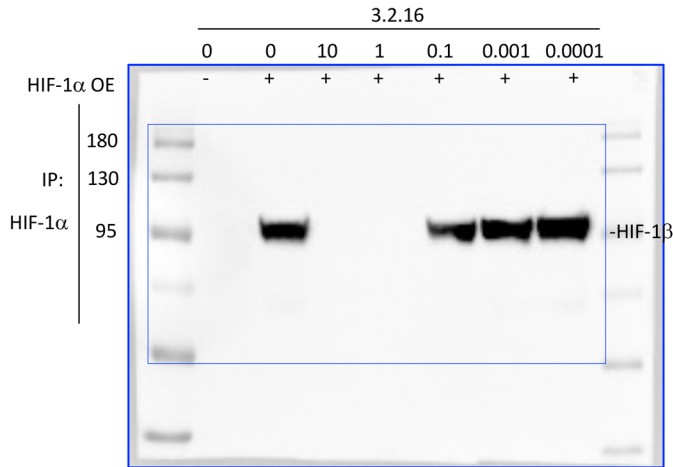

Figure 2D

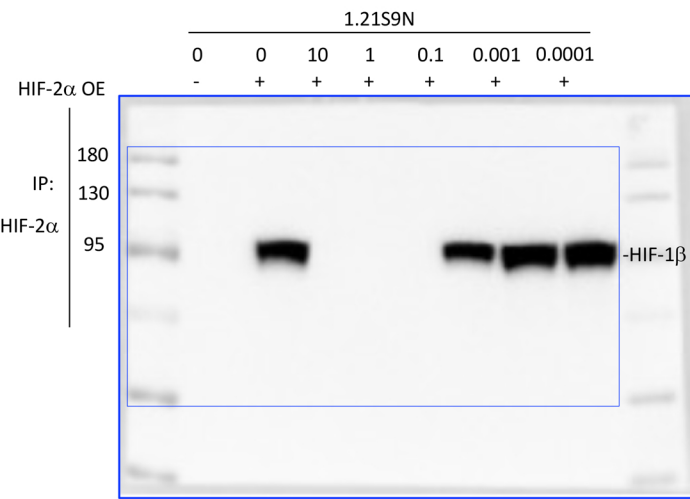

Figure 2F

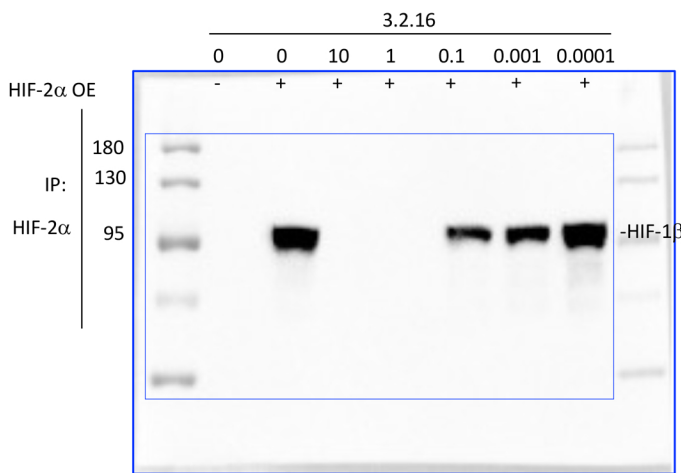

Supplement: SourceData F2 — is the source file for Fig. 2. [file jem_20251009_sourcedataf2.pdf]

Figure 4B

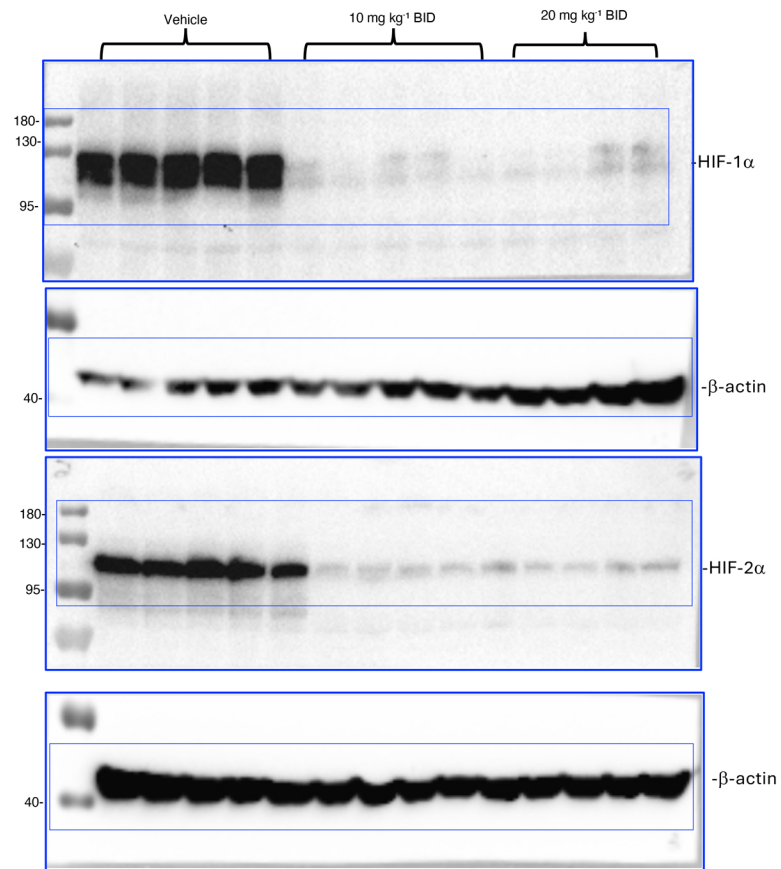

Figure 4E

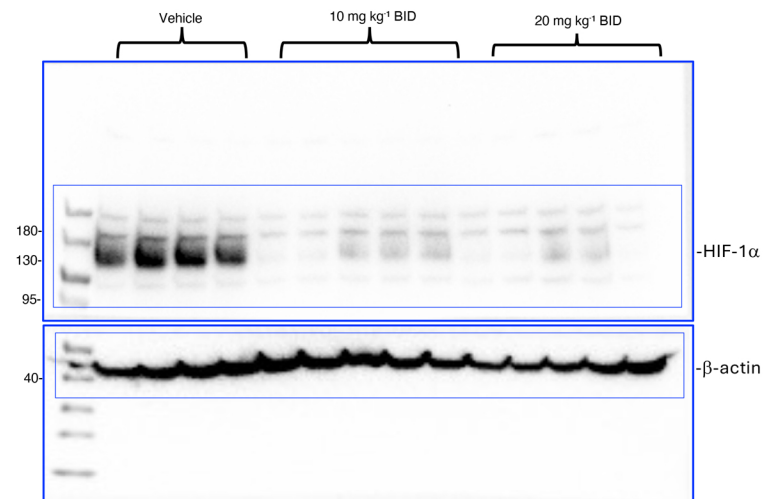

Supplement: SourceData F4 — is the source file for Fig. 4. [file jem_20251009_sourcedataf4.pdf]

Figure 5J

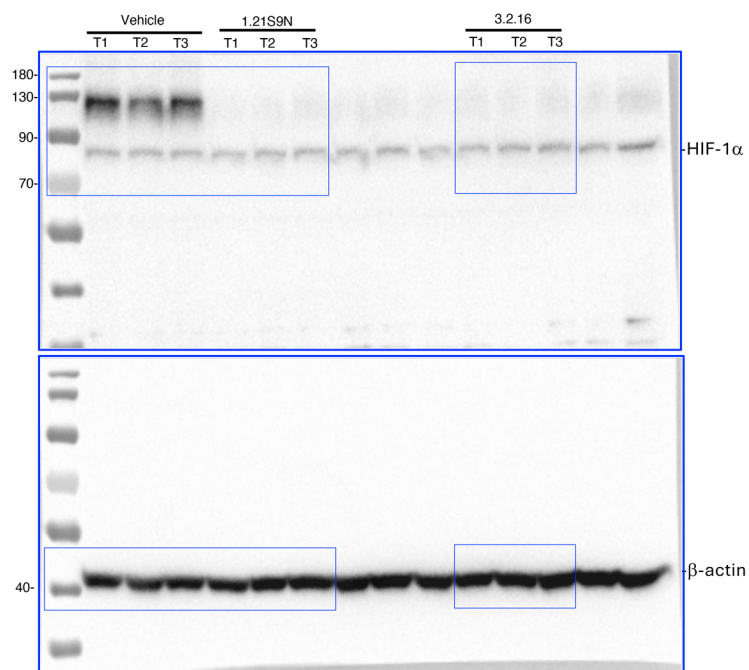

Supplement: SourceData F5 — is the source file for Fig. 5. [file jem_20251009_sourcedataf5.pdf]

Figure S3E

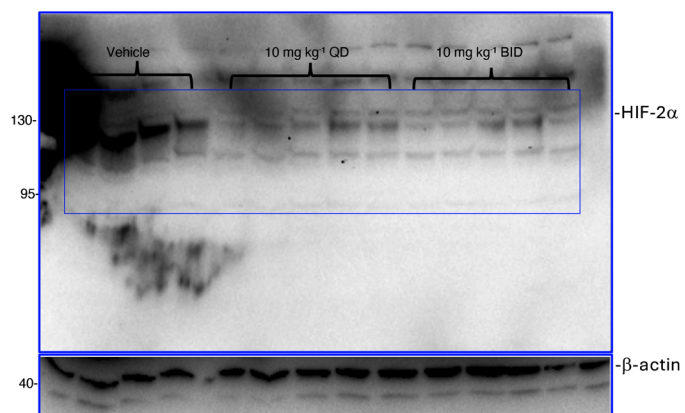

Figure S3K

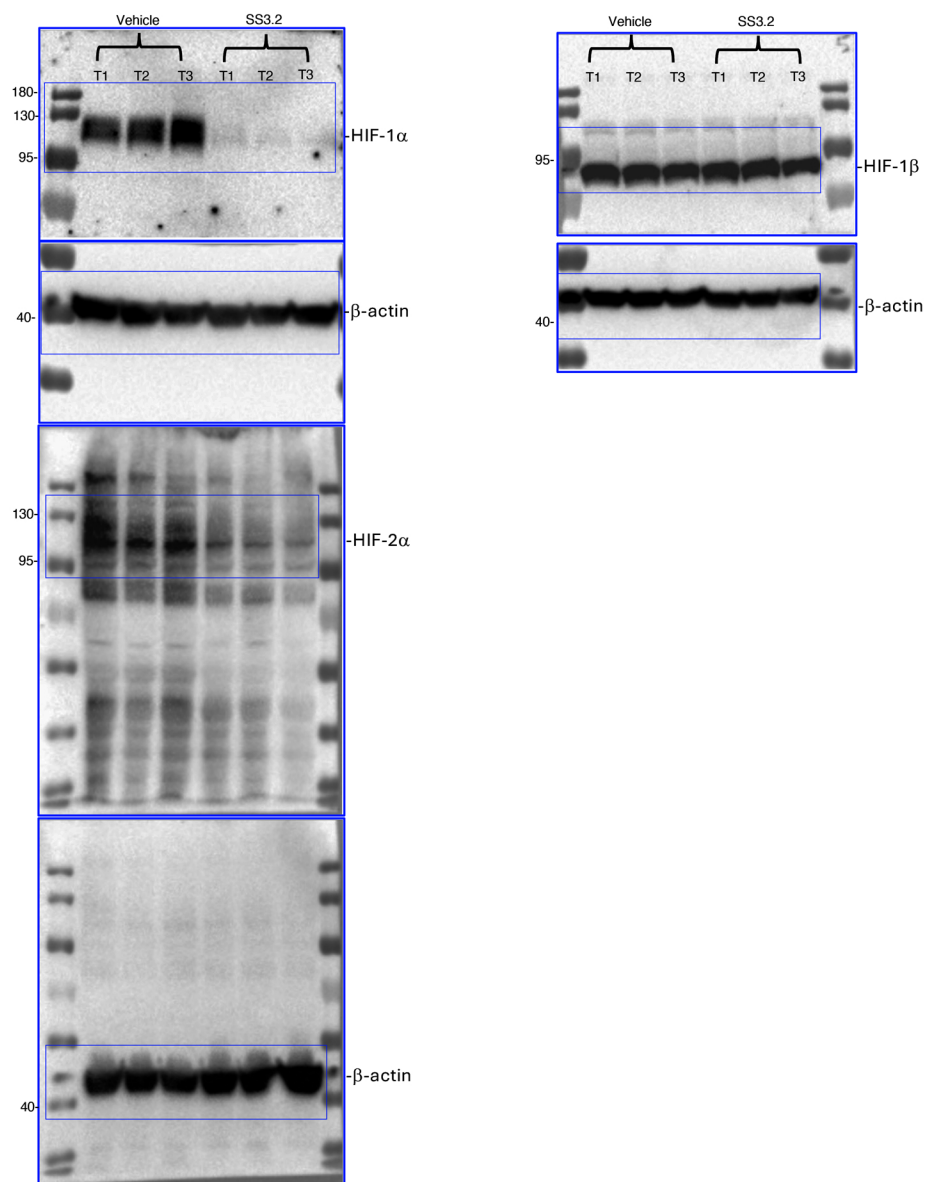

Supplement: SourceData FS3 — is the source file for Fig. S3. [file jem_20251009_sourcedatafs3.pdf]

Figure S5C

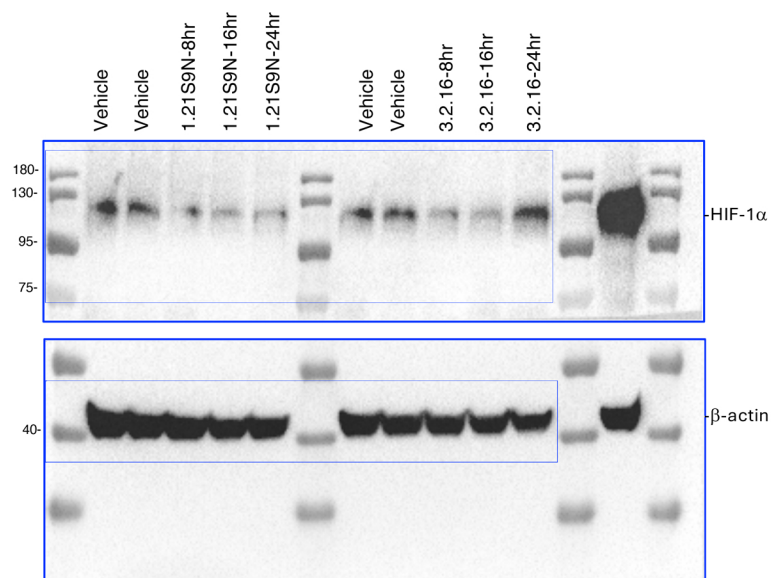

Supplement: SourceData FS5 — is the source file for Fig. S5. [file jem_20251009_sourcedatafs5.pdf]
