# Supplementary material for: Targeting conserved domains of hypoxia-inducible factors for cancer therapy
Source: J Exp Med. 2026 Apr 2;223(5):e20251009. doi: 10.1084/jem.20251009 (PMC13068195; doi:10.1084/jem.20251009)

Figure 3A

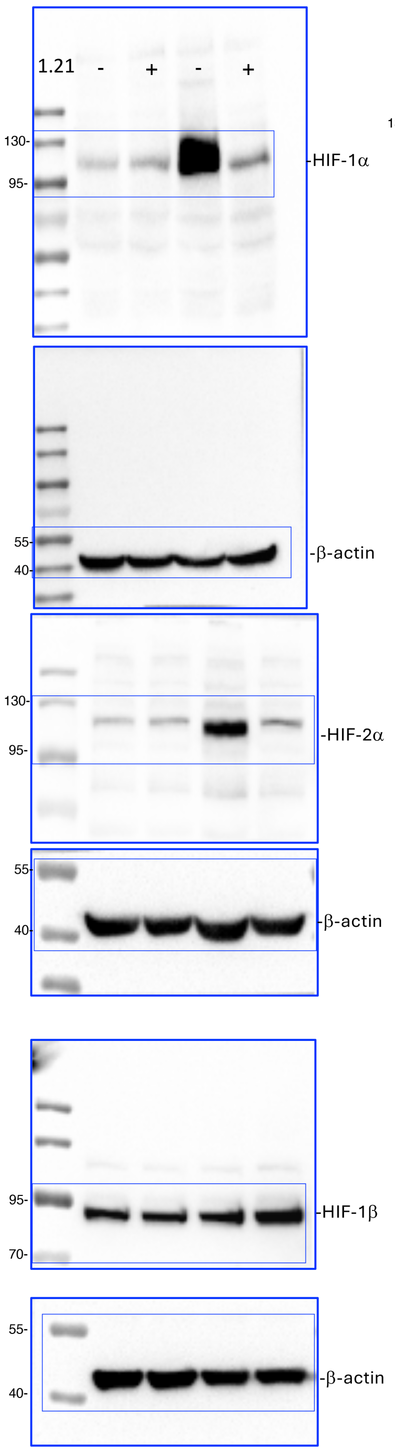

Figure 3B

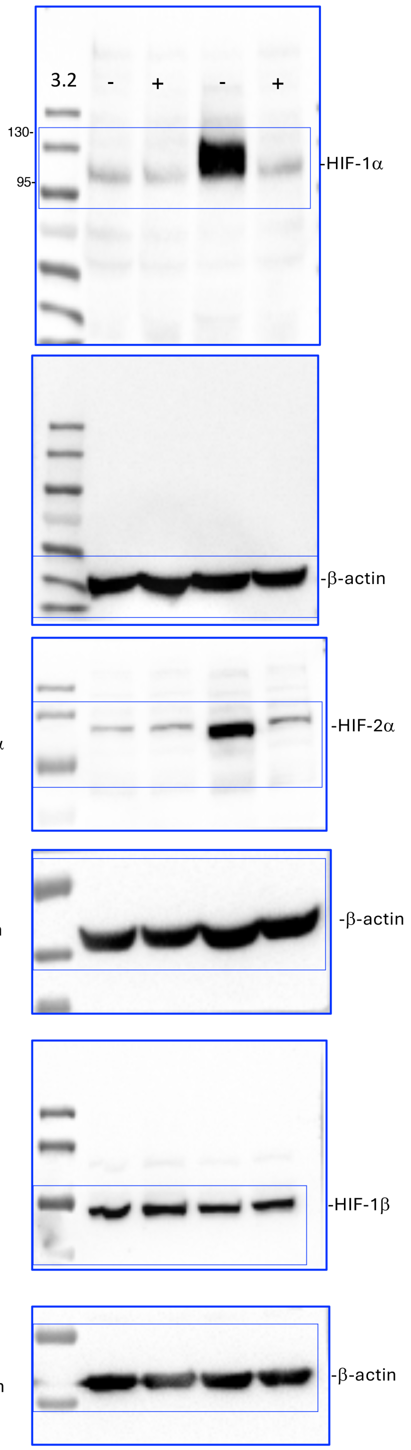

Figure 3C

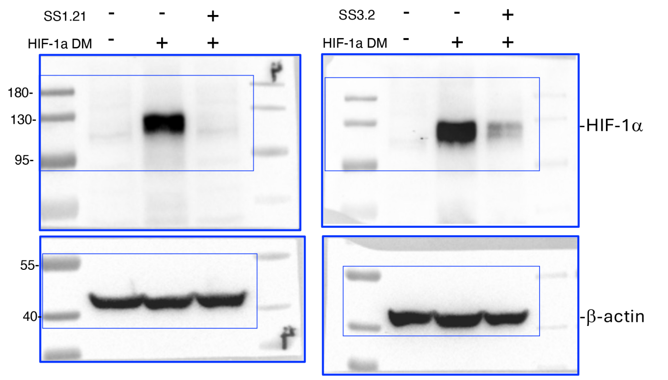

Figure 3D

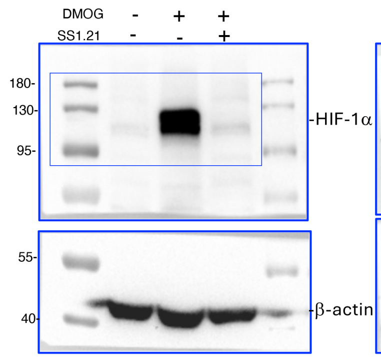

Figure 3E

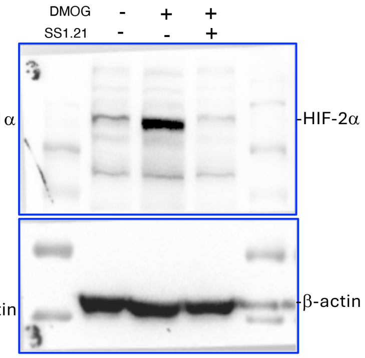

Figure 3F

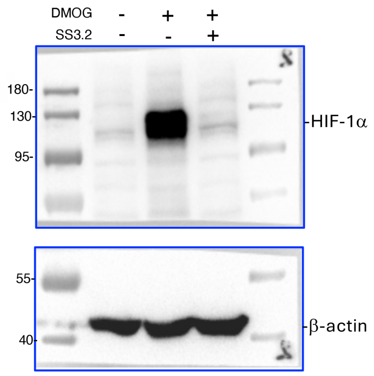

Figure 3G

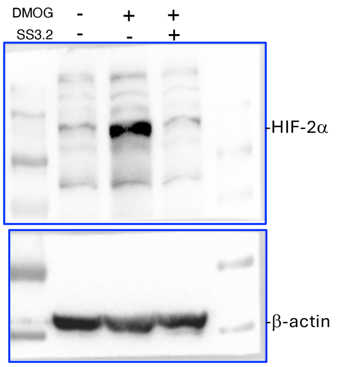

Figure 3H

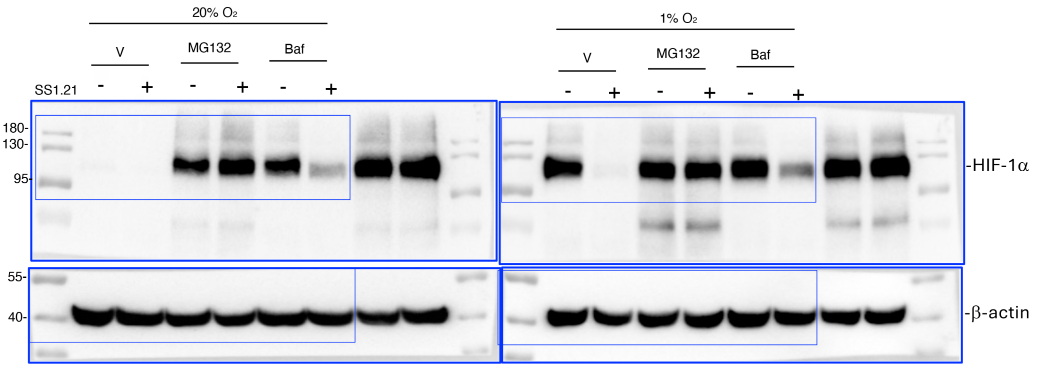

Figure 3I

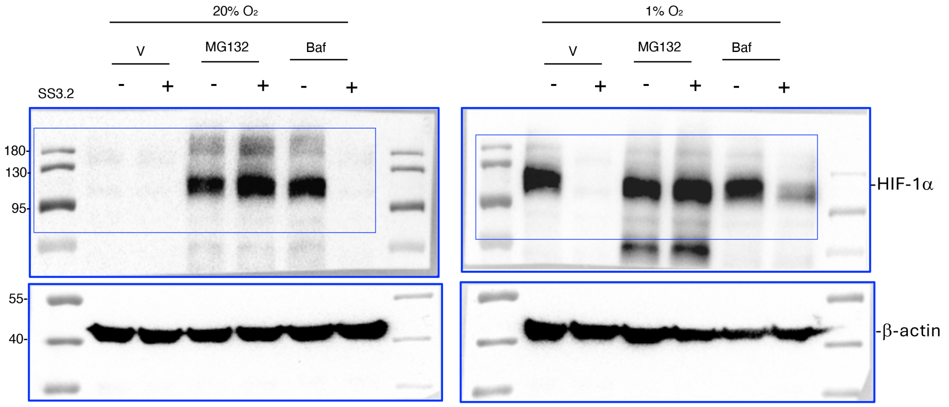

Figure 3J

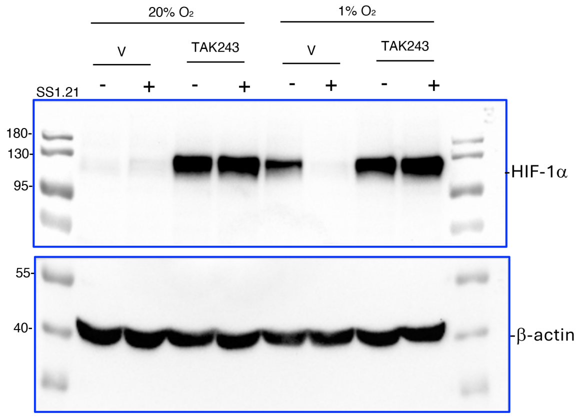

Figure 3K

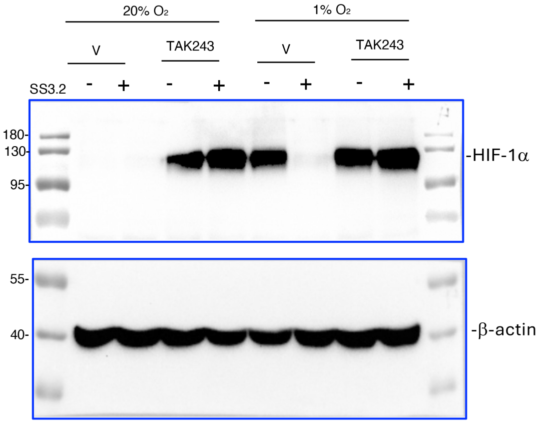

Figure 3L

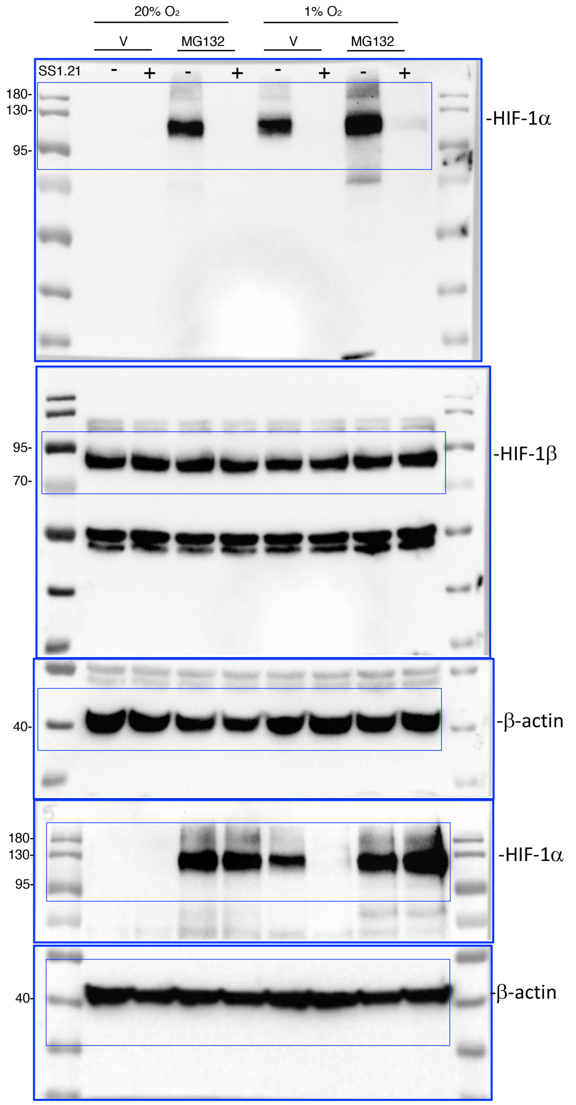

Figure 3M

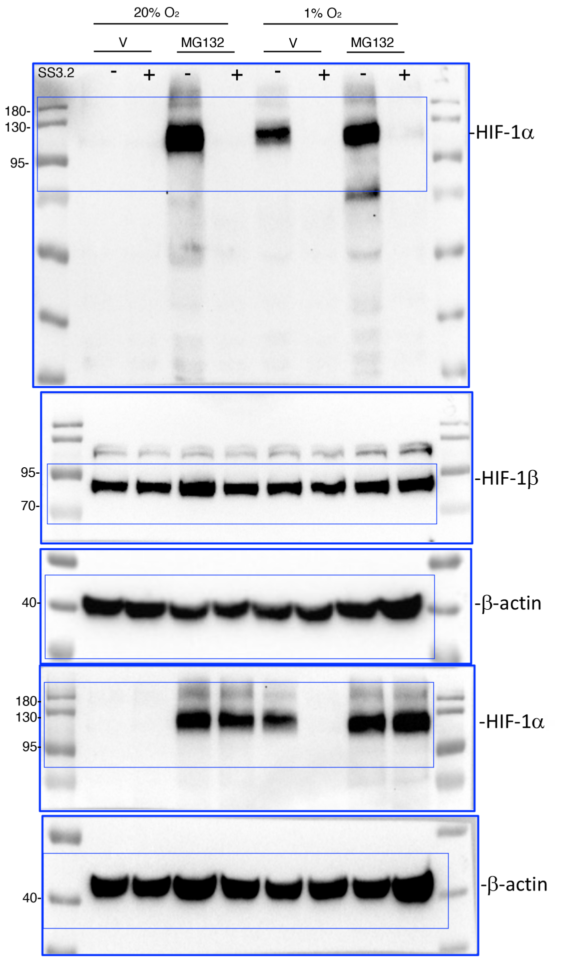

Figure 3O

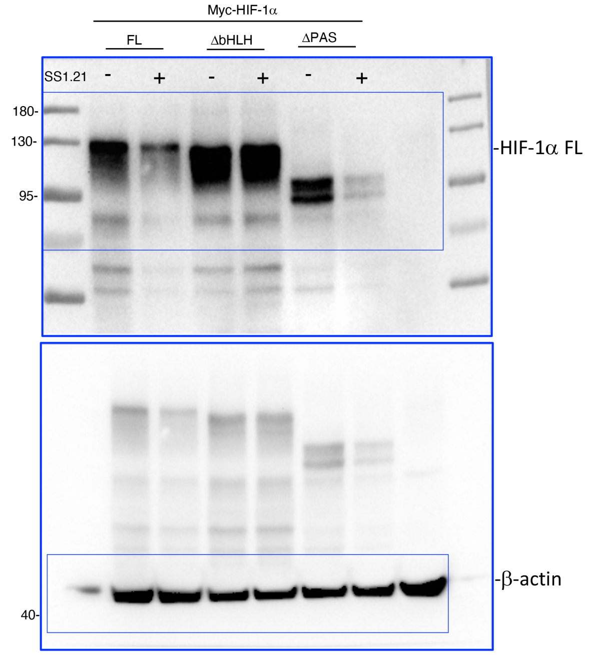

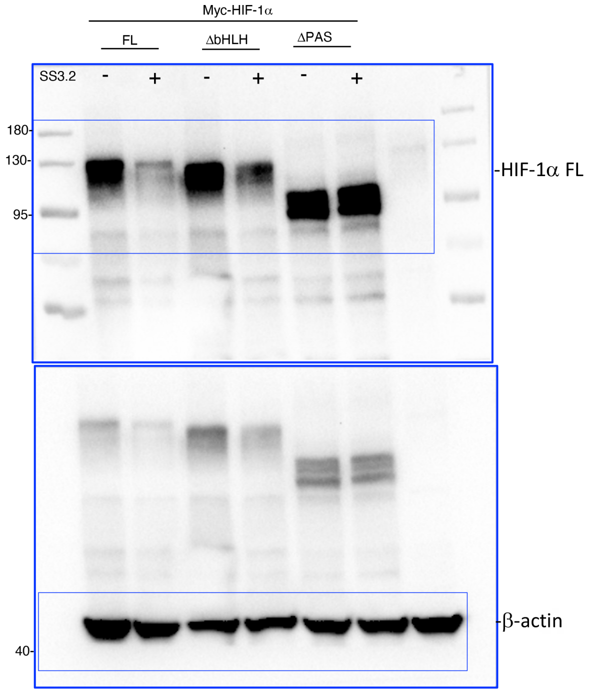

Supplement: SourceData F3 — is the source file for Fig. 3. [file jem_20251009_sourcedataf3.pdf]
